# Ultrathin Boron Growth onto Nanodiamond Surfaces via Electrophilic Boron Precursors

**DOI:** 10.3390/nano14151274

**Published:** 2024-07-29

**Authors:** Krishna Govindaraju, Tyanna Supreme, Daniel N. Labunsky, Nicole Martin, Juan Miguel Del Rosario, Alana Washington, Ezhioghode O. Uwadiale, Solomon Adjei, Sandra Ladjadj, Cynthia V. Melendrez, Sang-Jun Lee, Maria V. Altoe, Avery Green, Sebastian Riano, Sami Sainio, Dennis Nordlund, Abraham Wolcott

**Affiliations:** 1 Department of Chemistry, San José State University, 1 Washington Square, San José, CA 95192, USAtyanna.supreme@sjsu.edu (T.S.); juanmiguel.delrosario@sjsu.edu (J.M.D.R.); ezhioghode.uwadiale@sjsu.edu (E.O.U.); solomon.adjei@sjsu.edu (S.A.II);; 2 Linac Coherent Light Source, SLAC National Accelerator Laboratory, 2575 Sandhill Road, Menlo Park, CA 94025, USA; 3 Stanford Synchrotron Radiation Lightsource, SLAC National Accelerator Laboratory, 2575 Sandhill Road, Menlo Park, CA 94025, USAnordlund@slac.stanford.edu (D.N.); 4 The Molecular Foundry, Lawrence Berkeley National Laboratory, 1 Cyclotron Road, Berkeley, CA 94720, USA; 5 Covalent Metrology, 927 Thompson Pl, Sunnyvale, CA 94085, USA; 6 Microelectronics Research Unit, University of Oulu, Pentti Kaiteran Katu 1, Linnanmaa, P.O. Box 4500, 90014 Oulu, Finland

**Keywords:** diamond, nanoscale diamond, surface chemistry, boron neutron capture therapy, templated growth

## Abstract

Diamond as a templating substrate is largely unexplored, and the unique properties of diamond, including its large bandgap, thermal conductance, and lack of cytotoxicity, makes it versatile in emergent technologies in medicine and quantum sensing. Surface termination of an inert diamond substrate and its chemical reactivity are key in generating new bonds for nucleation and growth of an overlayer material. Oxidized high-pressure high temperature (HPHT) nanodiamonds (NDs) are largely terminated by alcohols that act as nucleophiles to initiate covalent bond formation when an electrophilic reactant is available. In this work, we demonstrate a templated synthesis of ultrathin boron on ND surfaces using trigonal boron compounds. Boron trichloride (BCl_3_), boron tribromide (BBr_3_), and borane (BH_3_) were found to react with ND substrates at room temperature in inert conditions. BBr_3_ and BCl_3_ were highly reactive with the diamond surface, and sheet-like structures were produced and verified with electron microscopy. Surface-sensitive spectroscopies were used to probe the molecular and atomic structure of the ND constructs’ surface, and quantification showed the boron shell was less than 1 nm thick after 1–24 h reactions. Observation of the reaction supports a self-terminating mechanism, similar to atomic layer deposition growth, and is likely due to the quenching of alcohols on the diamond surface. X-ray absorption spectroscopy revealed that boron-termination generated midgap electronic states that were originally predicted by density functional theory (DFT) several years ago. DFT also predicted a negative electron surface, which has yet to be confirmed experimentally here. The boron-diamond nanostructures were found to aggregate in dichloromethane and were dispersed in various solvents and characterized with dynamic light scattering for future cell imaging or cancer therapy applications using boron neutron capture therapy (BNCT). The unique templating mechanism based on nucleophilic alcohols and electrophilic trigonal precursors allows for covalent bond formation and will be of interest to researchers using diamond for quantum sensing, additive manufacturing, BNCT, and potentially as an electron emitter.

## 1. Introduction

Boron nanostructures and their allotropes have potential applications in a variety of areas, including optoelectronics, biological imaging, cancer therapy, and fuel combustion technologies. New synthetic pathways for nanostructured boron have seen a rapid increase in new synthetic pathways since the realization of borophene, the all-boron analog of graphene. Borophene was synthesized on Ag (111) surfaces and represents a significant accomplishment in nanoscale engineering of ultrathin materials [1,2,3]. Theoretical predictions [4,5,6,7,8,9] revealed that borophene should be metallic with a high level of anisotropy due to the unique electronic structure of boron, which was confirmed experimentally by Mannix and coworkers [1]. For spherical boron nanoparticles, the use of diborane gas with laser pyrolysis has been a standard technique, yet not one widely used because of the toxicity of the starting materials, high-powered laser systems, and vacuum chambers [10]. Researchers over the past decade have now diversified the modalities used for the synthesis of zero-dimensional boron nanomaterials, as reviewed by Yang [11]. New methodologies for boron quantum dots include the use of high energy sonication with exfoliation, hydrothermal reactions at 150 °C with elemental boron precursors, and mechanical milling processes with silica chemistry. Templating boron onto nanostructures has not been explored widely and could yield core-shell and one-dimensional nanostructures of increasing complexity. Here we use high-pressure high temperature (HPHT) nanodiamond (ND) as the templating material and find that alcohols on the diamond surface initiate growth.

Diamond has excellent physical properties, including high thermal conductivity (25 W/cm·K), extreme hardness (10 Mohs), a large optical bandgap (5.5 eV), a large electrical breakdown resistance (10 MV/cm), and chemical inertness. These physical properties could yield a robust template for layered structures yet reduced chemical reactivity and the atomic density of diamond surfaces present unique surface chemistry challenges that must be addressed for diamond-based technologies to mature. Currently, synthetic diamond is widely used in industrial settings, including in grinding, polishing, and diamond-tipped drill bit applications. Next generation quantum technologies with nitrogen vacancies and other color centers in diamond are emerging with demonstrations spanning photonics [12], quantum communication [13,14,15,16], quantum metrology [16,17,18], biosensing [19,20,21], electron-hole transport [22], and memory storage [23]. However, the all-carbon sp^3^ structure presents challenges in synthesizing uniform, covalently bound inorganic layers on diamond because of the high surface density of atoms (~22 atoms/nm^2^ on (110) and ~18 atoms/nm^2^ on (111)) and lattice mismatch. Synthesis of boron layers on diamond substrates is unexplored and presents a path to placing heteroatoms on diamond and examining the synthetic pathways for boron-diamond electronic or photonic materials. A previous study by Gavrilin and coworkers reacted diamond with boron halides and extracted activation energies for their reactions [24]. This study addresses several key questions motivated by density functional theory (DFT). Can diamond surfaces react with solution-based boron molecules to generate new covalent bonds? Does the boron-termination of diamond produce midgap states in the diamond electronic structure as predicted by DFT and yield a negative electron affinity surface (NEA)? [25,26].

Typically, boron is incorporated into the diamond lattice as a p-type dopant and produces conductivity in electrochemical and tribology settings [27]. NDs have been embedded in boric acid or B_2_O_3_ for neutron irradiation and generation of nitrogen vacancy centers (NV centers) by taking advantage of boron neutron capture processes [28]. Boron with three valence electrons, one less than carbon, has an inherent electron deficiency that produces an incredibly rich array of bonding motifs in the form of clusters, nanoparticles, and borophene with applications in medicine and energy applications [29]. Recently, carboranes have been utilized in drug design for their ability to instantly convert from a trigonal planar configuration (sp^2^) to a tetrahedral bonding structure (sp^3^) due to the electron-deficient nature of carborane complexes [30]. Advancements in the field of nuclear medicine have shown that carboranes are useful for boron neutron capture therapy (BNCT) [31]. Thermal neutron captured by ^10^B induces a fission reaction releasing lithium-7 (^7^Li) and alpha-particles (the nucleus of helium-4) along with gamma radiation that can be used to treat cancerous tissue. Currently, BNCT is used safely and effectively to treat brain, neck, and head cancers, yet it requires a neutron accelerator and extensive facilities [32,33]. For the therapy to be effective, an appropriate number of ^10^B atoms must be introduced to the neoplastic cells at an appropriate concentration, then irradiated after integration of the boron complex into the cell has been achieved. The most outstanding challenges for BNCT are the delivery of the boron complexes to the tumor site and a key hurdle that must be overcome to expand the use of BNCT for cancer treatment.

Here we demonstrate that alcohol-rich nanodiamond (ND-OH) acts as a template for boron overlayer growth via covalent bond formation and was characterized via overlapping microscopy and spectroscopy techniques. Electrophilic trigonal boron precursors react with 30–50 nm HPHT ND-OH samples at room temperature without catalysts in inert conditions. Vibrational spectroscopy confirmed a 75 cm^−1^ shift after the boron precursor reaction and was used qualitatively to determine the conversion rate. Electron microscopy revealed ultrathin layers of boron (~1 nm) and a marked change in morphology and size of the ND-B in parallel with elemental analysis. The boron reaction was discovered to self-terminate on the diamond surface and is thought to be limited by the number of alcohol groups, similar to an atomic layer deposition (ALD) type mechanism. Small boron signals using energy dispersive X-ray spectroscopy (EDS) made definitive elemental composition inconclusive due to the proximity of B Kα and C Kα emission peaks. Overlapping laboratory and synchrotron X-ray techniques were used to confirm C-O-B, B-B, and C-B bonding environments were present. A mechanistic model is also presented to explain the spectroscopic and microscopic observations during the boron reactions with ND-OH substrates. ND-B samples lacked solubility in dichloromethane (DCM), and a solubility study was performed to probe their dispersion properties. Boron bond formation on diamond can be leveraged by researchers to modify the surface dipole moment, charge density, and band-bending properties for biological labeling and quantum sensing applications with NV centers. Importantly, X-ray absorption spectroscopy (XAS) confirmed DFT predictions that midgap electronic states are present in ND-B samples and may suggest that the material may also have a NEA surface.

## 2. Materials and Methods

High-pressure high temperature nanodiamond powders (monocrystalline diamond powder, MSY 0–0.03 micron and MSY 0–0.05 micron) were purchased from Microdiamant, USA (Smithfield, PA). Anhydrous dichloromethane (≥99.8% Product #270997), boron tribromide (≥99% Product #230367), boron trichloride (1.0 M in methylene chloride #178934), and borane in tetrahydrofuran (#176192) were purchased from Sigma Aldrich (St. Louis, MO, USA). Boron carbide powder (95% 42 µm #815-96) and 400 mesh copper TEM grids with ultrathin lacey carbon film (#01824) were purchased from Ted Pella, Inc. (Redding, CA, USA). Boron powder (95% #47303) was purchased from Alfa Inorganics (Ward Hill, MA, USA). 4-inch silicon wafers coated with a 10 nm titanium adhesion layer and 100 nm of gold were purchased from LGA Thin Films, Inc. (Santa Clara, CA, USA). Spectra Tech, potassium bromide powder packets (#0016031) were purchased from Thermo Fisher Scientific (Waltham, MA, USA).

A Thermo Scientific (STF55346COMC-1) three-zone tube furnace was used to aerobically oxidize ~30 and 50 nm HPHT ND powders. Approximately 500–600 mg of ND powder was placed in a ceramic boat and inserted into the heating chamber, and a programmed heating cycle was started. Typical oxidation protocols include a 10 °C/min ramp to 525 °C and a hold of 525 °C for 5 h in open air conditions until a tan color is observed. Upon completion of the oxidation process, the NDs are then immediately placed in a glass scintillation vial and stored in a drying oven (~140 °C) to ensure a water-free diamond surface. ND-OH samples are alcohol rich and ready to be used for further boron chemistry.

Materials, including glassware, are placed in the 140 °C drying oven for 24 h prior to performing any water-sensitive syntheses to ensure complete removal of adsorbed water. Any additional materials, such as centrifuge tubes (50 mL polypropylene) and micropipette tips, were placed in a vacuum oven, set at 40 °C, 24 h prior to sample preparation. Trigonal boron molecules, such as boron tribromide and boron trichloride, are reactive with water, and all syntheses and purifications were performed in an inert nitrogen atmosphere glovebox.

**Figure 1:**
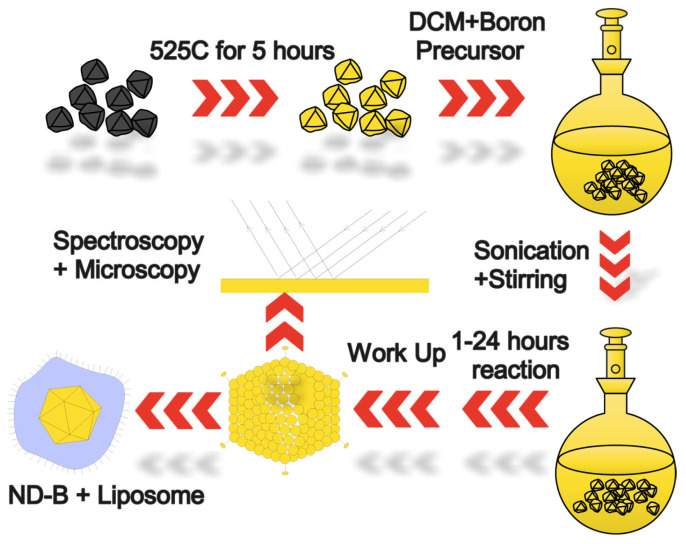
The schematic describes the workflow for the aerobic oxidation and boron chemical treatment of HPHT NDs in an inert atmosphere. Aerobic oxidation removes amorphous carbon and results in an alcohol-rich ND surface (ND-OH). Dry ND-OH is added to DCM and boron precursors and is briefly sonicated and stirred during the reaction. Purification via centrifugation and decantation (workup) is performed in an inert atmosphere. Characterization using overlapping spectroscopy and electron microscopy is performed, and ND-B is encapsulated in liposomes for a proof of concept for biomedical applications.

In a typical synthesis, 40 mg of ND-OH is placed inside a dried 100 mL round bottom flask and transferred into the inert atmosphere glove box. Boron tribromide (99%) and boron trichloride (1.0 M) in methylene chloride were reacted with ND-OH in 100 mL round bottom flasks. 40 mg ND-OH, 39.38 mL of anhydrous DCM, 0.62 mL BBr_3_, a stir bar, and a vacuum adapter were added, removed from the glovebox, and immediately the cup horn was sonicated. The BBr_3_ solution has a concentration of 161 mM for these reactions. For sonication, a cup horn sonicator (Fisher Scientific FB505) at 75% of its full power function of 500 W was used for two minutes to help solubilize the colloid. Following cup horn sonication, a bath sonicator is used to further solubilize the colloid at 40 °C for 10 min, and then the reaction is attached to a Schlenk line under inert conditions and allowed to stir at 300 rpm. Reaction times spanned 1–24 h at room temperature (See Figure 1).

**ND-B purification.** Post-synthetic workup was performed in the glove box with polypropylene centrifuge tubes and anhydrous DCM. Centrifugation at 5000 rpm for 25 min was performed until the trigonal boron-terminated NDs formed a pellet. The supernatant was decanted and analyzed via dynamic light scattering (DLS) to ensure minimal loss of ND-B. Resolubilization of the pellet in 10–15 mL of anhydrous DCM was followed by rigorous vortexing and bath sonication (outside of glovebox). Purification cycles are performed 3X using DCM. After purification, the ND-B samples were stored as powders or in solution in the inert atmosphere glovebox. Supernatants were analyzed via UV-vis absorption spectroscopy after the use of BBr_3_ and BCl_3_ to verify the molecular structure of the reddish and yellowish tinted DCM solution.

**ND-BCl_3_ Colloidal Solubility Study.** Purified ND-BCl_3_ powders were removed from the glovebox environment and dispersed in standard non-polar solvents and high-purity 18 MΩ water. Approximately 1 mg of ND-BCl_3_ powder was added to octane, toluene, octadecene, chlorobenzene, and water and briefly bath sonicated for 2–3 min. The dispersed colloid was then pipetted into a 1 cm × 1 cm quartz cuvette and studied by dynamic light scattering (DLS). The DLS is a Malvern Nano-S, and data was collected by using the following settings: Material = diamond with an index of refraction of 2.418, dispersant of octane→water, equilibration time for 10 s at 25 °C, and selection of a quartz cuvette. Measurements were performed with a run duration of 10 s, 10 runs, and 3 measurements. The histogram data as a function of nanoparticle diameter was imported into Excel, and a custom macro was used to transform and display the data. The transposed data was rendered in Igor Pro 8.0, and the 3 measurements were averaged, and Gaussian fits were applied using the Multi-Peak Fit 2.0 functions.

### Open Air and Inert Atmosphere DRIFTS

DRIFTS measurements were performed using the Harrick Praying Mantis DRIFTS attachment (DRK-3), high temperature reaction chamber (Harrick #HVC-DRM-5) and a Thermo Fisher FTIR (6700) equipped with an MCT/A detector. The high temperature reaction chamber was cleaned and stored in the vacuum oven prior to being brought back into the glovebox. OMNIC software (version 7.3) controlled the Thermo 6700 instrument. Temperature control of the DRIFTS chamber was controlled by Watlow EZ-Zone Configurator software (version 6). DRIFTS measurements were performed with 128 scans at a resolution of 2 cm^−1^ and background scans of near or equal signal intensity. KBr powder was stored in an oven (120 °C) for 24 h prior to being brought into the glovebox. All DRIFTS work was performed inside the glovebox under inert conditions. For collecting background data, 80 mgs of dried KBR were used to fill the DRIFTS cup completely and leveled to a flat surface. For collecting sample data, 3–4 mg of ND-OH or ND-BBr_3_ were added to 80–90 mg of KBr and mixed thoroughly. Sample DRIFTS data was collected in percent reflectance mode with a representative background scan.

**DRIFTS parameters and Analysis.** Kubelka-Munk transformations were performed individually with linear background corrections in Igor Pro software. Linear backgrounds were generated based on the averaged values of percent reflectance (raw data) in the DRIFTS regions of 2000–2200 cm^−1^ and 3800–4000 cm^−1^. This slope value was then applied to a y-intercept function (y = mx + b) and applied to spectra for normalized reflectance units (*R*) and then transformed using the Kubleka-Munk equation to make the data proportional to concentration:

(1) $$KM\;Units=\frac{\left(1-R^{2}\right)}{2R}$$

Infrared data sets were referenced to Infrared and Raman characteristic group frequencies: tables and charts by Socrates and HPHT ND and boron compound studies through the National Institute of Standards and Technology webbook [34,35,36,37,38,39].

**ND-OH and ND-B drop casting on Gold Coated Silicon Wafers for XAS and XPS.** Silicon wafers coated in gold from LGA thin films (Santa Clara, CA, USA) were cut into 1 × 1 cm^2^ squares, bath sonicated in isopropanol, acetone, and 18 MΩ water three times, and dried with a N_2_ gun. Note: A 10 nm Ti or Cr adhesion layer is used prior to 100 nm gold deposition on Si substrates. The wafers were cleaned of superfluous carbon via piranha solution (90 mL concentrated sulfuric acid and 10 mL hydrogen peroxide) for ~10 min in a crystalizing dish at 80 °C. Piranha-etched wafers were handled with Telon tweezers, rinsed thoroughly with 18 MΩ water, and dried with a N_2_ gun. After etching, the etched Au wafer was transferred to a 140 °C oven for 60 min and then transferred into the glovebox. In the glovebox, 200 µL of 1 mg/mL ND solution in anhydrous DCM was deposited via micropipette, and the DCM evaporated while partially covered by a crystallizing dish. Additional Au-coated etched wafers were also dried for 24 h at 140 °C prior to introduction into the glovebox to ensure a water-free surface. Oxidized and boron-coated nanodiamond samples were also prepared using 18 MΩ water as a solvent under open-air conditions at 1 mg/mL concentrations in a similar fashion on a hotplate set at 50 °C.

**Synchrotron XAS Measurements.** X-ray absorption (XAS) measurements were performed at beamlines 8-2 and 10-1 at the Stanford Synchrotron Radiation Lightsource, SLAC National Accelerator Laboratory, using a spot size of <1 mm^2^. All samples were handled in an inert atmosphere glovebox and mounted to an Al sample bar with conductive carbon tape (#16073-4 Ted Pella, Inc., Redding, CA, USA). The ND-B, ND-OH, and control samples were transported to the beamline in a sealed polypropylene jar, and a magnetic mounting piece was attached to the sample bar in an inert atmosphere glovebox. The sample bar was transferred to the beamline and purged with N_2_ gas for 90 min inside a plastic glove bag attached to the transfer chamber. Once purged, the transfer chamber was vented, and the sample bar was introduced into the transfer chamber under a positive pressure N_2_ flow. Samples were pumped down in the transfer chamber to 1 × 10^−7^ torr and introduced to the analysis chamber with a nominal pressure of 5 × 10^−9^ torr.

XAS was measured in total electron yield (TEY) mode using 42 × 42 μm, 40 × 40 μm, and 30 × 30 μm slits for C1s, B1s, and O1s edges, respectively. TEY mode probes approximately 5–10 nm of the sample depth, and all experiments were conducted under ultrahigh vacuum conditions (~5 × 10^−9^ torr). X-rays are focused with optics, while the reference absorption intensity of the incoming X-ray beam was measured using a sample of gold-coated mesh and used to correct for beam instability. All XAS data was collected at an incident electric field vector of 54.7°. XAS spectra were treated with a linear pre-edge background subtraction from a region before the absorption edge of boron, carbon, and oxygen at 180–190 eV, 260–280 eV, and 510–530 eV, respectively. Post-edge atomic normalization was also performed in the continuum region at 340 eV for carbon and 580 eV for oxygen and performed using a batch processing macro in Igor Pro. Energy calibration was performed using the signal from the diamond core-hole exciton, which is determined to be 289.0 eV as described elsewhere [40]. Energy calibration of the synchrotron light source was performed during grating changes with a Ni slab (Ni L3 absorption) and a 1-point fitting procedure.

**XPS Measurements and Analysis.** A Thermo Scientific X-ray photoelectron spectrometer (XPS), a K-Alpha Surface Analysis instrument, at the Molecular Foundry was utilized to probe for carbon, boron, and oxygen on the surface of the ND-OH and ND-B samples. The K-Alpha Plus XPS has a combined low energy electron, ion flood source and is utilized to suppress charging during all data collection. The X-ray source and detector are an Al Kα micro-focused monochromator and is equipped with a 180° double hemispherical analyzer with a 128-channel detector, respectively. Low resolution and high-resolution pass energies were set to 200.0 eV and 50.0 eV, respectively. Low resolution and high-resolution energy step sizes were 1.0 eV and 0.1 eV, respectively. The electron acceptance angle was 55°, and survey scans were performed over a binding energy range from 0–1350 eV with a pass energy of 200 eV. Three scans were summed, and a dwell time of 10 ms was used. ND-B samples that required air-free transfer from the glove box to the K-alpha XPS instrument were loaded into the inert atmosphere transfer module. ND-B samples were loaded into the transfer module for analysis and transported from SJSU to The Molecular Foundry (Lawrence Berkeley National Laboratory, Berkeley, CA, USA).

XPS analysis and data rendering were performed using Igor Pro software (version 6.3) and the CASA-XPS software package (version 8.0) with standard background subtraction and fitting protocols. In a standard method, a linear background subtraction was performed on B1s high resolution scans in Igor Pro, and then peaks were fit to a Voigt line shape with a mixed Gaussian and Lorentzian contribution. Peak widths were typically held to a FWHM of 1.5–2.0 eV as appropriate for the spectral features. CASAXPS software was used for quantitative analysis of survey scans to determine the atomic percentage of individual elements, and Tougaard backgrounds were applied with relative sensitive factors (RSF) values being applied for each element. RSF values for B, C, and O were 1.0, 1.8, 2.93, and 2.8, respectively. Percentage atomic concentrations ($X_{A\;}$) are calculated using the following equation:

(2) $$X_{A}=\frac{\left(I_{A}E^{\alpha })/{(R}_{A}T(E)\right)}{\sum \left(I_{i}E^{\alpha })/(R_{i}T\left(E\right)\right)}$$

wherein *X_A_* is the atomic percentage of element *A*, *I_A_* is the intensity of the element *A*, *R*_A_ is the RSF factor for element *A*, *T*(*E*) is the transmission function of the instrument fat kinetic energy (*E*), and in the denominator are all elements summed with the appropriate intensities and RSF factors (*I_i_* and *R_i_*) [41]. The alpha term in the exponent of kinetic energy *E* is used to adjust for analyzer specifications. The VAMAS (Versailles Project on Advanced Materials and Standards) file collected by the K-Alpha instrument allowed for all needed transmission function information to be applied for quantification purposes, and the VAMAS file type is ISO 14976 compliant [42]. Table 1 contains the inelastic mean free paths and RSF factors for boron, carbon and oxygen K-edge photoelectrons.

## 3. Results and Discussion

### 3.1. Morphology Changes after Boron Chemistry on ND Cores and Limitations of EDS Mapping to Confirm Boron

**Figure 2:**
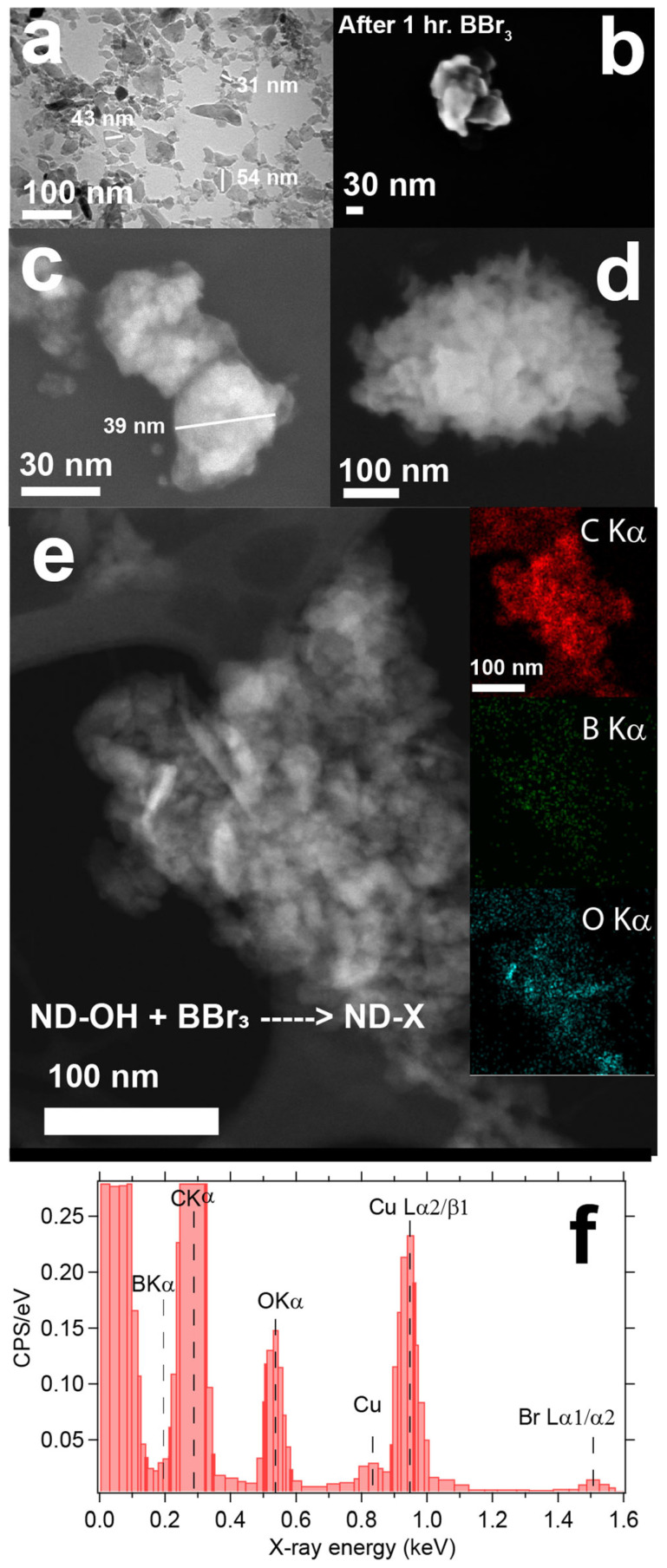
TEM and EDS data of ND-OH samples reacted with BBr_3_ for 1 h. Ultra-thin layers of an unknown identity on HPHT NDs are examined with electron microscopy before and after reactions with boron precursors. Changes in morphology, size, and evidence of electron transparency are apparent. The HPHT NDs have a shard-like appearance from ball milling and range in size from 20–80 nm (**a**). After BBr_3_ chemistry for 1 h, the ND-X become rounded, coated, and crosslinked (**b**). Collections of ND-X range in size from 10 s to 100 s of nm as seen in panel (**c**,**d**). Energy dispersive X-ray spectroscopy (EDS) was also used to map the presence of boron, carbon, and oxygen, yet B Kα and C Kα X-rays overlapped spectrally and were inconclusive (panels **e**,**f**). Residual bromine is also observed at ~1.5 keV and confirms that trace B-Br bonds exist.

Immediate observations of BBr_3_ or BCl_3_ reactions with ND-OH were the generation of a reddish or yellowish tint in the supernatant, and UV-visible absorption measurements were consistent with Br_2_ and Cl_2_ formation. The generation of Br_2_ and Cl_2_ as side products will be commented upon in the mechanism section, yet it is important to state that we have built a picture of the diamond-boron chemistry. The initial technique to probe the ND-OH samples after boron chemistry was via high resolution transmission electron microscopy (HRTEM) and energy dispersive X-ray spectroscopy (EDS). HPHT NDs are generated by ball milling of single crystal diamond and lead to the preferential cleavage of the diamond {111} facets, a shard-lifke appearance, and a bulk-like alcohol-rich surface after aerobic oxidation [42,43]. Figure 2 outlines the stark transition that ND-OH undergoes after chemical treffatment with BBr_3_, from the shard-like appearance of HPHT NDs after aerobic oxidation to the rounded morphology and clustering with an unknown surface coating obscuring the NDs (Figure 2a–f). The unknown layer on the ND surfaces (ND-X) is electron transparent, and the ability to view both the thin layers and underlying ND is evident (Figure 2c,d). In panel 2c, a ~39 nm ND-X particle is obscured by the overlayer, and a diffuse material is observed blanketing the underlying diamond core. Differentiation between the diamond core and overlayers with SEM and TEM is nontrivial, and determination of the identity and thickness of the layered structure was needed. Confirmation that the underlying material is diamond was achieved with electron energy loss spectroscopy (EELS), and the data is provided in the supporting information (Figures S1 and S2).

Point analysis and scanning EDS were performed to map the elemental composition of ND-X samples by tracking the B Kα, C Kα, O Kα, and Br L X-ray emissions. The results showed strong signatures for carbon and oxygen and weak signals for boron (Figure 2e inset). The locations of the C Kα and O Kα signals, highlighted in red and cyan, respectively, are uncontroversial and spatially map to the cluster location in Figure 2e. In contrast, the spatial correlation between the B Kα and C Kα signals must be treated skeptically due to their proximity in energy, the large C Kα intensity, and the EDS energy resolution of 130 eV (Figure 2f). Challenges in identification and quantification of boron are highlighted in our study, as the core material is 100% carbon and the ultrathin layer suspected to be boron could not be definitively identified with EDS. Carbon signals overwhelm the small contributions from boron; a strong C Kα signal can be improperly integrated as B Kα counts. Higher in energy, the Br Lα1/α2 signals were also detected in the EDS full area scan at ~1.5 keV and gave partial evidence of the BBr_3_ reaction with the ND-OH surface (Figure 2f). ND-OH, elemental boron powder, and boron carbide (B_4_C) control samples were also analyzed by SEM/EDS to highlight B Kα and C Kα peak positions and to quantify elemental composition as a function of electron accelerating voltage (see Figures S3–S6).

### 3.2. Starting ND-OH Vibrational Structure Consistent with Alcohols and a Surface Free of Adsorbed Water

**Figure 3:**
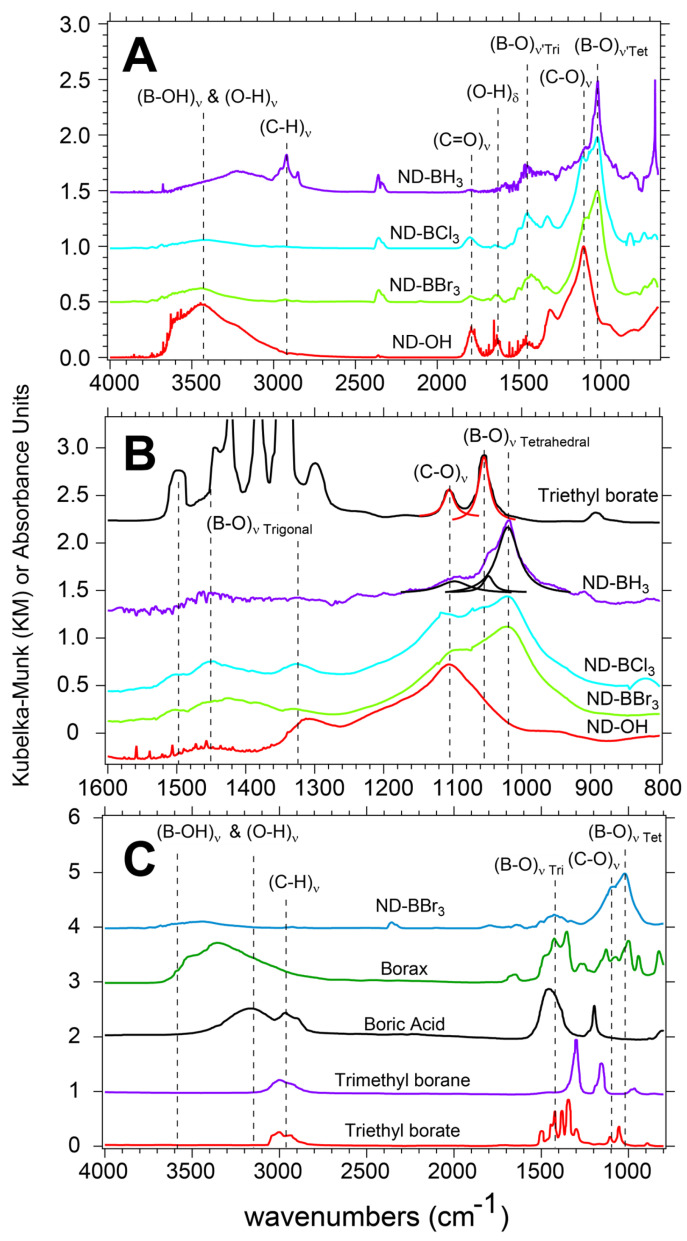
DRIFTS data of boron-coated ND samples and controls show a mix of trigonal and tetrahedral bonding environments. A clear shift in the alcohol peak of ND-OH at 1105 cm^−1^ is seen after boron reactions are complete (**A**). BBr_3_, BCl_3_, and BH_3_ precursors reacted with ND-OH and yielded new features in the fingerprint region due to new C-O-B bonds at 1250–1550 cm^−1^ and 950–1200 cm^−1^ for trigonal and tetrahedral bonding, respectively (**B**). Triethyl borate as a control shows (B-O)_ν’Tri_, (C-O)_ν’,_ and (B-O)_ν’Tet_ modes, and when compared to the ND samples, it shows that a majority of boron centers are in the tetrahedral bonding structure and a minority remains trigonal (**C**). A comparison to various boron controls shows the general trends in the B-OH region and the fingerprint region in comparison to ND-BBr_3_. Borax shows the greatest vibrational activity in the (B-O)_ν’Tet_ region and is consistent with the peaks observed ~1025 cm^−1^ in ND-B samples.

Vibrational spectroscopy was next used to probe the surface structure of the NDs after EDS was found to be unconclusive. HPHT ND powders (ND-OH) are produced by aerobic oxidation in a tube furnace, dispersed in DCM, and readily reacted with electrophilic boron precursors to form ultrathin shells in a glove box environment and on a Schlenk line. When oxidized at high temperature in open air conditions, 30–50 nm ND particles are purified of adsorbed amorphous carbon, yielding a tan powder that is hydrophilic and has an alcohol-rich surface [44]. Diffuse reflectance Fourier infrared transform spectroscopy (DRIFTS) was used to analyze the surface vibrational modes of the ND-OH powders before and after the boron chemistry. Post-aerobic oxidation yields a significant peak at 1105 cm^−1^ corresponding to the (C-O)_ν_ surface bonds of alcohols, in concert with (O-H)_δ_ bending at 1640 cm^−1^, as well as a broad O-H stretch from 3000–3500 cm^−1^, both due to adsorbed water. A minor carboxylic acid peak at 1785 cm^−1^ was observed, and past observations measured a maximum of 7% surface coverage of acids on HPHT ND surfaces [45]. When focusing on the 1640 cm^−1^ peak and 3000–3500 cm^−1^ region after boron chemistry, the majority of (O-H)_ν_ and all of (O-H)_δ_ intensity are reduced due to removal of adsorbed water (see Figure 3A). Temperature programmed desorption DRIFTS showed the desorption of water and complete elimination of the 1640 cm^−1^ peak in previous work [46]. Because the starting reactants (trigonal boron molecules) are sensitive to water and oxygen, especially the boron trihalides, the adsorbed water peak at 1640 cm^−1^ is used to quantify water desorption from our sample prior to a reaction.

#### Trigonal and Tetrahedral Boron Centers on ND Surfaces Post Reaction

ND-B samples were found to have vibrational features of both trigonal and tetrahedral boron bonding environments and were dependent on the boron electrophile used. There are 2 boron-oxygen asymmetric stretching regions and are denoted by their position as either a trigonal ((B-O)_ν’Tri_) or tetrahedral (B-O)_ν’Tet_ in the 1250–1550 cm^−1^ and 950–1200 cm^−1^ range, respectively. DRIFTS spectra of ND-OH reacted with BBr_3_, BCl_3,_ and BH_3_ show peaks at 1105 cm^−1^, 1055 cm^−1^, and 1021 cm^−1^ which are assigned to the (C-O)_ν_, (B-O)_ν’Tet1_ and (B-O)_ν’Tet2_ surface bonds, respectively (Figure 3A). The (B-O)_ν’Tet_ vibrational modes are 32 cm^−1^ apart, and a previous FTIR study of boric acid at neutral and basic pH conditions is consistent with our assignments [39]. DRIFTS spectra of BBr_3_, BCl_3_, and BH_3_ treated ND-OH samples show contributions from (C-O)_ν_, (B-O)_ν’Tri_, and (B-O)_ν’Tet_ surface bonds and indicate that these moieties simultaneously occupy surface sites. BH_3_ reacted samples showed the greatest contribution from (B-O)_ν’Tet_ surface groups and a small contribution from (C-O)_ν_, indicating an elimination of alcohols from the surface. An unusual outcome of BH_3_ treated samples was the emergence of (C-H)_ν_ mode due to hydrogenation of the ND surface. The carboxylic acids appear to be reduced by BH_3_, as expected, and may also rapidly react to form organoborates, which rationalize the limited number of alcohols on the ND-BH_3_ surface [36]. In contrast, BBr_3_ and BCl_3_ reacted samples did not show that hydrogenation occurred and instead showed more signal and structure in the (B-O)_ν’Tri_ region, while acids were generally unaffected. Vibrational modes for B-Br or B-Cl were not observed, yet the presence of B-Br was detected using thermogravimetric analysis-mass spectroscopy.

Quantifying the conversion of alcohols to organoborates on the HPHT ND surface with DRIFTS data is difficult and will be avoided; instead, qualitative conclusions based on NIST control spectra are used. For clarity in assigning vibrational features, tetrahedral and trigonal modes are in the 1000–1100 cm^−1^ and 1200–1600 cm^−1^ range, respectively. In Figure 3B,C, a series of boron molecules, including sodium tetraborate decahydrate (Borax) and triethyl borate (TEB), are displayed to illustrate the vibrationally active modes for trigonal and tetrahedral bonding environments. FTIR of TEB helps to frame the results and discussion due to the presence of both C-O and B-O bonds being present in its molecular structure. TEB has trigonal (B-O)_ν’Tri_ modes in the range of 1250–1550 cm^−1^, while (C-O)_ν_ is observed at 1105 cm^−1^ and (B-O)_ν’Tet_ at 1055 cm^−1^. Borax with 2 trigonal and 2 tetrahedral boron centers shows added vibrational complexity, yet the range for trigonal and tetrahedral virbational modes is consistent. As observed in Figure 3B, BH_3_ reacted samples show little (B-O)_ν’Tri_, a reduction in (C-O)_ν_, and a large contribution from (B-O)_ν’Tet_ at 1055 cm^−1^. BBr_3_ and BCl_3_ reacted ND samples in contrast have increased (B-O)_ν’Tri_ resonance and comparitively larger (C-O)_ν_ is observed. These results indicate that in inert conditions, the halogenated compounds tend to retain their trigonal bond structure in comparison to the BH_3_-reacted samples. Lorenztian fits of the TEB and BH_3_ data are used as a guide to show contributions in the 1000–1100 cm^−1^ region. The presence of (B-O)_ν’Tet_ in TEB is understood to be due to degradation of the sample yielding a small contribution to the FTIR spectra. Borax shows intense absorption from the (B-O)_ν’Tet_ modes and is clearly seen in Figure 3C. Quantitification of the alcohol to organoborate conversion is ongoing and will be addressed using complimentary techniques in the future. To verify the above assignments, complimentary surface-sensitive techniques are required, as described in the next sections.

### 3.3. XPS Reveals Boron Templated ND Samples Contain B-O, B-B, and B-C Bonding Environments

**Figure 4:**
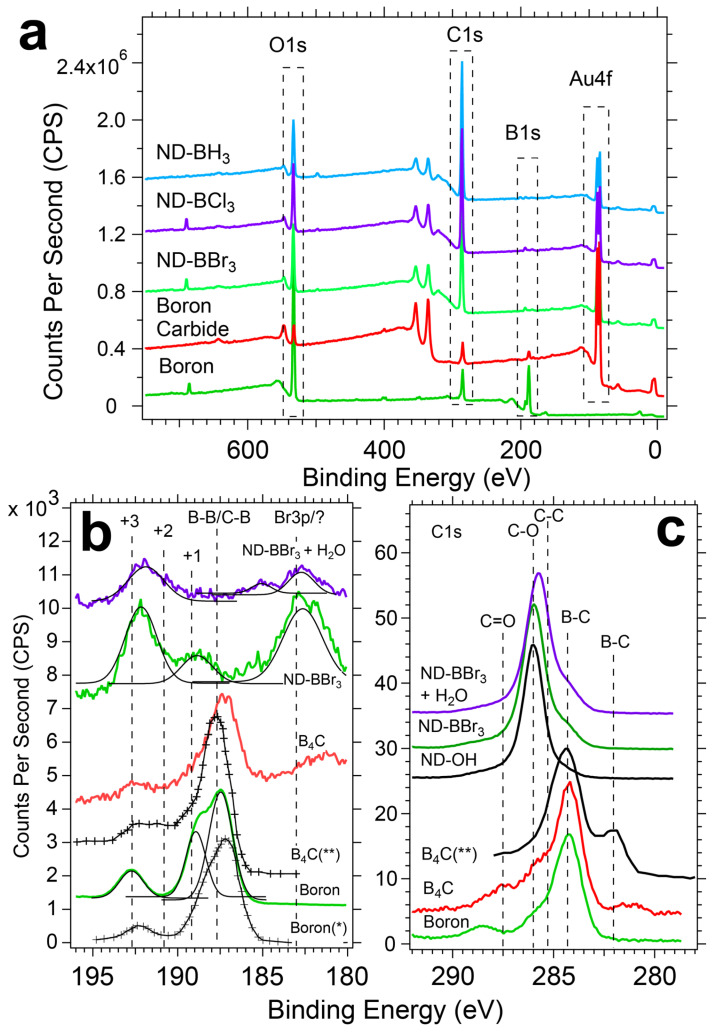
XPS data shows the small contribution of the B1s signal to both survey and high-resolution scans after ND-OH reactions with boron precursors. Control samples of boron carbide (C_4_B) with oxidation layers and elemental boron provide evidence that ND-BH_3_, ND-BCl_3_, and ND-BBr_3_ are largely terminated with organoborates. Boron oxidation states are dominated by +2 and +3 states, while ND-BH_3_ does contain a weak B1s signal in a +1 state at 190.3 eV. Shifts in B1s peak location due to Br, Cl, and H bonding to boron centers complicate the interpretation with B-O bonding environments.

XPS provides surface-sensitive and element-specific spectra that confirm the ultrathin layering of boron and a mixture of boron bonding environments. Due to the inconclusive presence of boron during EDS analysis, it was essential to identify and quantify boron present on the ND surface through other techniques. XPS survey scans were used to quantify the elements using the CASAXPS software package with Versailles Project on Advanced Materials and Standards (VAMAS) data files (see Figure 4a). As a base line value, ND-OH samples typically have carbon and oxygen compositions of ~85% and ~15%, respectively. Because of the inelastic mean free path of photoexcited electrons, the contribution of C1s electrons from ND cores will decrease as overlayers are grown [47].

**Summary of Quantitative XPS of elemental boron, B_4_C and boron treated NDs.** The unique air-free reactions with trigonal boron precursors generated B1s% atomic concentrations in the range of ~1–3%, and decreases were seen with age and exposure to water. Empirically, BBr_3_ reactions with ND-OH were more synthetically successful and were explored more frequently. Because these reactions were exploratory and had no precedent, we frame the XPS assignments based on control samples containing only carbon and boron with oxidized surface species. The summary of % atomic concentrations is given in Table 2 and will be discussed here. When quantified, elemental boron powder and boron carbide (B_4_C) controls have B 1s, C 1s, and O 1s% atomic concentrations of 57.0%, 15.3%, and 27.7% and 30.0%, 52.1%, and 17.9%, respectively. As delivered, elemental boron powders had the expected oxygen termination and significant carbon contamination from the manufacturing process and adventitious carbon. B_4_C showed non-stoichiometric contributions from carbon with additional oxygen signal from the boron oxide-terminated surface. Of the boron-treated ND samples, ND-BBr_3_ samples showed the largest average B1s contribution that ranged from ~1.5–3.5%, ND-BCl_3_ ranged from ~0.6–2.8%, and BH_3_ from ~0.8–2.0%. Oxygen content varied widely from ~6–28% across the ND-B samples, and the increase in O 1s signal beyond 15% is correlated with the reaction of water vapor or O_2_ with the boron terminated surface. Observed decreases in O 1s would be consistent with the etching of the diamond surface, reductive chemistry, or a decrease due to the boron overlayers. For example, ND-BBr_3_-1 and ND-BBr_3_-2 are the same sample examined 1 month apart (see Table 2). ND-BBr_3_-1 has B1s and O1s content of 3.1% and 6.5%, respectively, and after 1 month the values changed to 2.5% and 27.7%. The likely source of oxygen is contamination of ND-BBr_3_ from residual O_2_ or water in the glovebox environment, trace water in the anhydrous DCM, or exposure to the atmosphere during transport to the XPS facility. Because trigonal boron precursors are good electrophiles and highly sensitive to water, it is concluded that boron centers on ND-B samples are chemical sinks for residual oxygen.

**XPS of elemental boron and B_4_C.** The boron bonding environment on the ND surface is heterogenous and contains signatures of 3 chemical species, including B-O, B-B, and B-C, and requires a comparison to elemental boron and B_4_C (see Figure 4b). First, let us examine the B 1s signatures from elemental boron powder from this study and boron (*) from Moddeman [48]. Unannealed and untreated elemental boron have B-B bonds, (B-O)^+1^ and (B-O)^+3^, observed at 187.5 eV, 189.0 eV, and 192.7 eV, respectively. Moddeman and colleagues used high temperature annealing that removed the oxide species (B_x_O_y_) or B_2_O_3_, and the annealing resulted in a single feature at 187.5 eV representative of B-B bonds. Next, untreated B_4_C (**) from Jimenez et al. has 4 features for B-B/B-C, (B-O)^+1^, (B-O)^+2^, and (B-O)^+3^ located at 187.7 eV, 189.1 eV, 190.6 eV, and 192.6 eV, respectively [49]. The Ted Pella supplied B_4_C powder was used as delivered and has a peak at 187.3 eV, a shoulder at 188.6 eV, and 192.7 eV, representative of B-B/B-C, (B-O)^+1^ and (B-O)^+3^, respectively. These peak positions represent the best metric to determine the boron bonding in the ND system, and these control XPS spectra are well matched to previous work.

**XPS Signatures of a Heterogenous boron bonding on the ND surface.** The ultrathin boron layers presented challenges with laboratory XPS due to small signals and energetic overlap with species including bromine. In the region of 185–195 eV, there are 2 overlapping regions for the B1s and Br3p photoemission peaks. B1s are observed in the region of 188–194 eV, while Br 3p_3/2_ and 3p_1/2_ span 182–190 eV based on the large spin-orbit splitting of ΔE = 6.6 eV with a peak area ratio of 2:1. B 1s XPS of ND-BBr_3_ in DCM (green trace) has peaks at 182.6 eV, 188.9 eV, and 192.1 eV that are assigned to Br 3p_3/2_ and 3p_1/2_ and boron in the (B-O)^+3^ state. The bromine bonding environment is not Br^−^ (peak near 181.8 eV), nor C-Br observed at 183.8 eV as found in brominated graphene [50], and is likely bromine bound to boron as B-Br, though C-Br is possible. The (B-O)^+3^ can be understood as an isolated boron center bridging 3 oxygen atoms and retaining a trigonal planar bonding structure. An alternative bonding structure includes a single C-O-B surface bond and subsequent oxidation of the boron center with residual water or oxygen similar to a boric acid molecule in a trigonal or tertrahedral bonding environment, consistent with our DRIFTS results. Dispersion of ND-BBr_3_ in water with sonication (purple trace in Figure 4b) results in the B1s decreasing in intensity, and peak shifts occur. After linear background substractions of the ND-BBr_3_ samples, a difference spectrum reveals losses of counts at 181.8 eV, 183.2 eV, and 188.6 eV and gains at 185.2 eV and 190.7 eV based on gaussian fits. Surprisingly, a 182.8 eV peak remains post-water exposure after a 50% reduction in intensity, while there is no complimentary Br 3p peak at 189.4 eV (see Figure S8 in Supplementary Materials). These observations suggest the post-water exposure peak at 182.8 eV could be a stable bromine bound to carbon (C-Br) or not related to bromine. The new peak at 185.2 eV is also not expected and is not consistent with bromine. Qunatitative XPS analyses of boron and bromine before and after water exposure are 1.2%/0.93% and 0.19/0.02%, respectively. Boron % atomic concentrations reduced by 23%, as bromine was reduced by 90%, which would be the case when bromine is bound as B-Br and is readily hydrolyzed to HBr and discarded during purification cycles. A similar mechanism for hydrolysis of the boron shell to B(OH)_3_ would justify the observed 23% reduction. In order for B 1s photoelectrons to have lower binding energy than ~187.5 eV (typical for B-B), an electron donating group or structure would be required, as seen with B 1s features at 185.4 eV with an azide present [51]. N-type nitrogen dopants present in the diamond host (100–200 ppm) are a plausible electron-donating source, yet further work is required to clarify this observation.

C1s XPS of ND and control samples reinforces the heterogous bonding environment on the diamond surface, which includes C-O and C=O bonds, and a new feature at 284.3 eV assigned to direct boron-diamond bond ormation. Unpurified boron has a large carbon content with a prominent peak at 284.7 eV due to adventicious carbon from the manufacturing process, with 2 features at 286.2 eV and 289.0 eV assigned to C-O and C=O bonds, respectively. Untreated B_4_C from our measurements and B_4_C from Jiménez showed 2 main features at 282.0 eV and 284.3 eV. Jiménez explained the 282.0 eV and 284.3 eV peaks are due to the C-B-C chain and carbon in the icosahedron of B_4_C, respectively (Figure 4c) [52,53]. The alcohol-rich ND-OH starting material has features for the sp^3^ C-C core of the diamond, C-O and C=O at 285.4 eV, 286.2 eV, and 287.5 eV, respectively. Comparison to ND-BBr_3_ in DCM and after water exposure spectrum highlights the emergence of a shoulder at 284.3 eV that agrees well with the B-C_icosa_ peak of B_4_C. There were no C 1s features found at 282 eV for CBC chain bonding. These results indicate that a minority of the diamond-boron bonding environment is similar to the B_11_C icosahedra found in B_4_C powders in conjunction with organoborates (C-O-B) bonds at 192 eV. Direct diamond lattice-boron bond formation was not expected, yet the planar boron precursors would be well suited to quench sp^2^-like Pandey chains and receive electron density from the diamond surface similar to addition across an alkene in classic organic chemistry [54]. The retention of the B-C_icosa_ peak after water exposure indicates that the bond is stable and resistant to hydrolysis, similar to benzoxaboroles [55].

**Figure 5:**
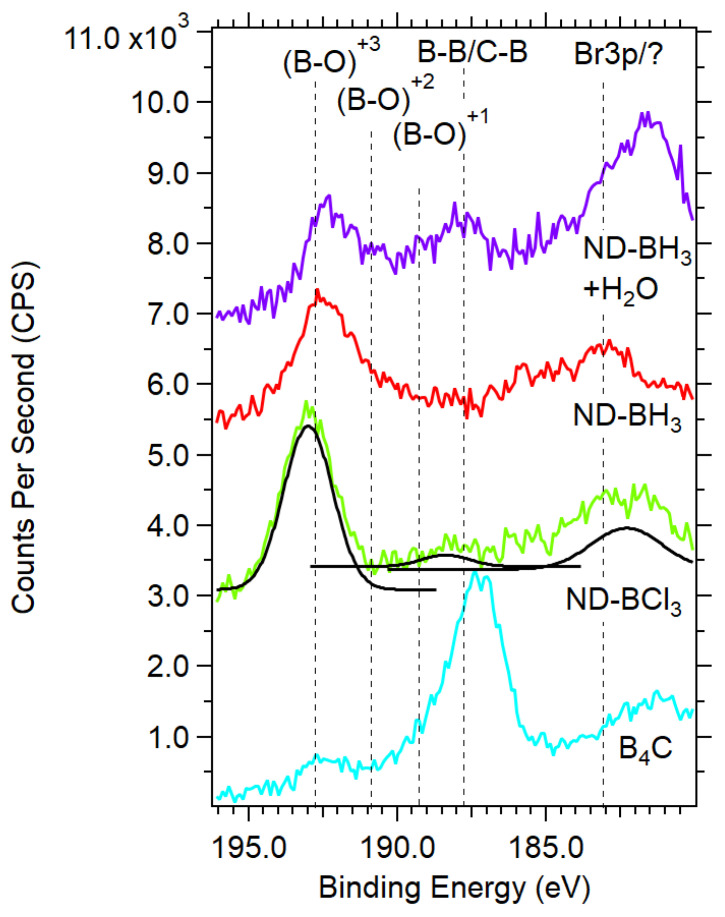
B1s XPS spectra of samples before and after dispersion in water and subjected to sonication. Samples are as notated: Exposure to water and sonication of ND-BH_3_ results in a decrease of over 50% in boron termination and (B-B/B-C) bonding at 188.9 is observed after water exposure while an anomalous peak at 182–183 eV arises. ND-BH_3_ and ND-BCl_3_ samples have the (B-O)^+3^ peak, which is assigned to a boron center bound to 3 surface oxygen atoms.

ND-OH samples reacted with BCl_3_ and BH_3_ have features consistent with the C-O-B bond formation in concert with lower energy B1 features. ND-BCl_3_ and ND-BH_3_ have 2 broad features at 183 eV and 193.0 eV representative of an unknown source and (B-O)^+3^, respectively (see Figure 5). The main peak of ND-BCl_3_ at 193.0 eV confirms the general C-O-B bond formation via nucleophilic attack of the alcohol on the boron center. A 183 eV peak is an anomaly and cannot be attributed to Br 3p since no such Br source is present. ND-BH_3_ also contains the main C-O-B peak attributed to the (B-O)^+3^ oxidation state of boron with a weak feature at 183 eV. Exposure of ND-BH_3_ to water yielded 2 significant changes, with the emergence of a large peak at 181.5 eV and another at 187.9 eV. The 187.9 eV peak is consistent with B-B or B-C bond formation, while the 181.5 eV peak is not well understood. The anomalous peaks at 182–184 eV are not due to a Si plasmon from the gold-coated substrate due to the lack of Si signal, and a Si plasmon is very broad from 163–185 eV. The emergence of the B-B/B-C peak is consistent with two mechanisms of forming a layered boron sheet or a reductive reaction that removes oxygen from the diamond surface. The formation of a B-B peak during the dispersion of ND-BH_3_ in water could be caused by the removal of hydrogens from B-H as H^+^ and the formation of new boron-boron bonds in plane with the diamond surface. Unlike the halide-leaving groups generating HCl or HBr, protons in BH_3_ would likely decompose and off-gas H_2_. Here we highlight that the B1s signal is low and remains low regardless of reaction time (~1–24 h), which argues that a self-terminating mechanism similar to atomic layer deposition is plausible. A mechanistic perspective will be discussed later in this letter.

**Figure 6:**
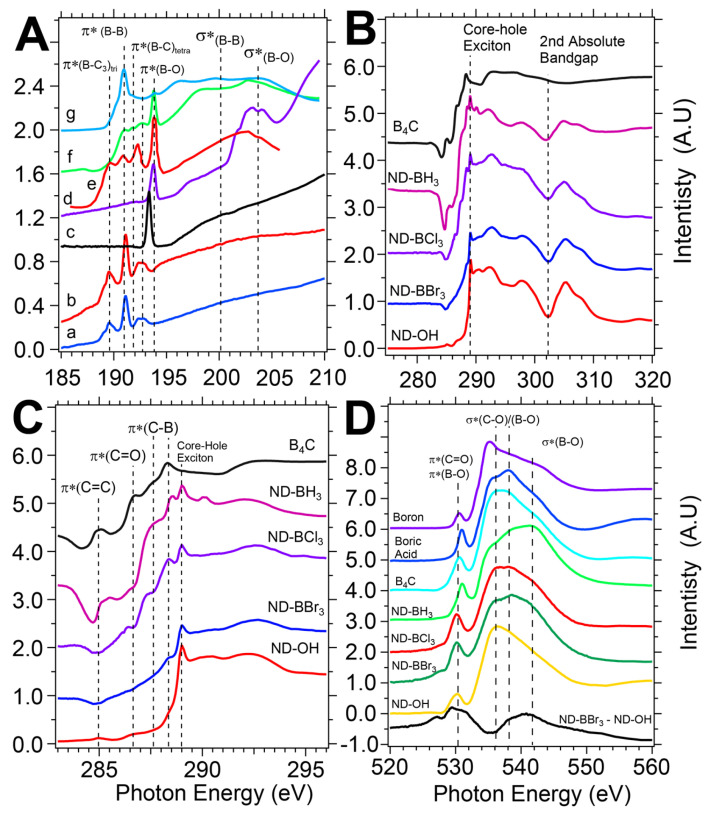
B1s and C1s XAS data reveals a complex bonding environment for control samples and ND-BX_3_ samples. B1s XAS data in panel (**A**) includes ND-BBr_3_ (a), ND-BCl_3_ (b), ND-BH_3_ (c), B(OH)_3_ (d), BC_3_ thin film from Caretti et al. [56] (e), boron powder (f), and B_4_C (g). Boron-treated ND samples have new C1s pre-edge features attributed to new carbon-boron bonds on the diamond surface (panel **B**). The pre-edge area shows the emergent unoccupied states and agrees well with carbon-boron bonds in B_4_C (panel **C**). O1s edges show moderate changes in the bonding environment of oxygen on the diamond surface including an increase in delocalized π*(B-O) resonances around ~530–531 eV after boron reactions (**D**).

**B1s X-ray absorption spectroscopy reinforces a boron-diamond electronic structure similar to B_4_C and BC_3_ thin films.** Synchrotron-based X-ray absorption spectroscopy (XAS) is element-specific, probes both the surface and subsurface environment (~10 nm), and directly maps the unoccupied density of states based on selection rules (∆l=±1). B1s XAS revealed that boron chemistry generated new unoccupied electronic states within the diamond bandgap and discovered that B-B, B-C, and B-O moieties are present. Typically, B1s features are categorized by π* states below 195 eV, while σ* states are above 195 eV, following the usual convention. The new boron-layered diamond surface required a comparison with control samples that included B(OH)_3_ (boric acid), boron powder, and B_4_C (boron carbide), and these features will be discussed here (see Figure 6). B(OH)_3_ has a simple spectra dominated by the π*(B-O) peak at 193.8 eV and σ*(B-O) resonances in a range of 201–206 eV. Boron in the +3 oxidation state has a large 193.8 eV peak while boron suboxides (B_x_O_y_) have a 192.8 eV peak, also assigned to π*(B-O) resonances. As delivered, amorphous boron powder has an absorption onset at 189.5 eV and peaks at 191.1 eV, 191.8 eV, 192.8 eV, and 193.8 eV. The π*(B-B) resonances are observed at 191.1 eV and 191.8 eV and are resolved clearly in our study, whereas some previous studies show a smooth shoulder leading to the large 193.8 eV peak. Both boron oxide peaks are also clearly resolved at 192.8 eV and 193.8 eV and assigned accordingly. B_4_C, made of B_11_C icosahedron linked by C-B-C chains, is the sole control sample with boron-carbon bonds [56,57]. The onset of absorption begins at 189.3 eV and leads to the largest resonance at 190 eV in B_4_C, but its origins are not clear. Jiménez describes the peak as orginating from bonding between boron icosahedra through boron-boron bonds or the C-B-C chain that links the icosahedra [53]. The B1s transitions from the region of 188–194 eV will be expanded upon to describe the heterogenous bonding environment of ND-B samples, and BC_3_ and a-B_2.5_C thin films are used for comparison [56,57].

The B1s XAS spectra provided an in-depth probing of the covalently bound boron layers on the diamond surface, with evidence of sp^2-^trigonal B-C bonds being present. First, let us inspect the ND-BBr_3_ sample with an onset of absorption at 188.9 eV and a series of π* states reaching 194 eV. ND-BBr_3_ and ND-BCl_3_ contain 5 features: a shoulder at 189 eV and peaks at 189.6 eV, 191.1 eV, 192.4, and 192.8 eV (Figure 6A samples (a/b)). Peak assignments are based on the control samples and a BC_3_ thin film mentioned previously by Caretti and Jiménez. The 189 eV and 189.6 eV resonances overlap well with the BC_3_ and a-B_2.5_C thin films and can be explained by a disordered or amorphous trigonal bonding environment and labeled π*(B-C_3_)_tri_ [56,57]. No reference samples match these low energy peaks, and this report would be the third observation of such low energy resonances. The dominant peak at 191.1 eV is well matched to the main B_4_C peak and is assigned to the π*(B-B) resonance, which reinforces the formation of a boron-rich layer. To reinforce the spectral overlap, the BC_3_ thin film from Caretti has been added to the B1s spectra as sample (e) and described previously as peak B_1_ [56]. A smaller resonance at 192.4 eV is cautiously assigned as π*(B-C_3_)_tetra_ and originally described as a boron-rich carbide feature by Jiménez and later Caretti (peak B_2_ in Caretti) [53]. A peak at 192.8 eV is less controversial and is understood as the suboxide of boron and assigned as the π*(B-O) resonance. Notably, the large π*(B-O) peak at 193.8 eV is absent and suggests that the 192.4 eV peak is representative of the pristine diamond lattice C-O-BX_2_ bond without water or oxygen contamination. ND-BH_3_ (sample c) has properties more closely resembling those of B(OH)_3_ or B_2_O_3_, with a large π*(B-O) peak at 193.4 eV and broad σ* resonance from 196–202 eV. Because the features are indicative of exposure of the sample to water and oxygen either from trace water in the anhydrous DCM or during transport to SSRL, we conclude that a boric acid termination has been created on the ND surface. A reexamination of ND-BH_3_ will be conducted in future reports.

**C1s XAS confirms new diamond midgap electronic states are generated.** When examining the electronic structure of ND-BX_3_ samples, we observe new mid-gap states introduced by the boron chemistry that can be clearly seen with C1s spectroscopy. A classic ND-OH spectrum contains all features of a single crystal diamond sample with minor features at 285 eV and 286.7 eV due to trace primal sp^2^ carbon or Pandey chains and carboxylic acids with assignments to the π*(C=C) and π*(C=O), respectively [58,59]. The diamond core-hole exciton peak and 2nd diamond bandgap are seen at 289.0 eV and 302 eV, respectively, and are reflections of the diamond core material (Figure 6B) [40]. Post boron chemistry drastically modifies the pre-edge features below 289 eV, as compared to the B_4_C control sample. The fine features are displayed in Figure 6C. Dips in the pre-edge region around 285 eV are caused by adventitious carbon on the reference current channel used for beam normalization and, while undesirable, do mildly affect the C1s observations for ND-BH_3_ only. Because diamond has a large bandgap of 5.5 eV, the surface termination can introduce states that can act as electron acceptors or donors, and we recommend the work of Petit and Sangtewesin for further background [60,61].

DFT calculations of clean and oxygen-terminated 100 diamonds layered with boron atoms predicted the results observed with XAS [25,26]. Sun et al. and Shen et al. describe in great detail the bonding environment that includes C-B, B-B, and C-O-B termination, the introduction of midgap states caused by boron, and transitions from positive to negative electron affinity surfaces. While the HPHT NDs are 25–50 nm and terminated largely with 111 facets, the above DFT work supports the XAS results well. Shen outlines the adsorption of boron on an oxygen-terminated surface (ethers and ketones) and shows the oxygen sites reorganized with boron adsorption, including the breaking of C-O-C bonds to form a C-O normal to the diamond surface. Boron adsorption energies (ΔE_ea_) were found to be a function of surface coverage, with a 1 monolayer (ML) coverage producing the most stable structures, yielding ΔE_ea_ = 5.47–5.89 eV/atom and the production of B-B bonds (please see Figure 6 and Table 3 from Shen [25]). They also report that at 1 ML coverage, the surface retains a PEA surface and may stabilize the NV center in the −1 charge state. Conversely, the adsorption of boron on clean or bare diamond yields a NEA surface at 1 ML coverage. Sun et al. extended those results and show B-B chains forming across the surface in a zigzag and dimer-like configuration. 3 bonding structures described as 1 ML-α, β, and γ with bridging and boron-boron chains were found with electron localization functions showing covalent bond formation between adjacent boron-boron sites. Density of states calculations in both studies show new states in the diamond bandgap ranging from 1.6–3.66 eV above the valence band minimum and qualitatively agree with the broad absorption onset we see in our C1s XAS studies described below.

An important aspect of the chemistry and XAS spectroscopy relies on the trigonal boron precursors containing no carbon atoms. Therefore, any new carbon bonding environments generated reside at the diamond surface, and electron delocalization may play a role due to boron. Jiménez rationalized the C1s features of B_4_C by comparison to closo-carboranes (C_2_B_10_H_12_), and we will do the same here. Closo-carboranes are a molecular cage structure with 2 carbon atoms at ortho, meta, and para positions and are an analogue of the icosahedra found in the B_4_C structure [62]. Recall that boron is electron deficient, with 3 valence electrons bonding in clusters or sheets being delocalized and is exemplified with boron producing 5 nearest neighbor bonds in C_2_B_10_H_12_. Boron clusters tend to form planar and 2-D structures due to delocalized σ and π bonding, and the unique bonding is described as fluxionality, or the rapid degenerate rearrangement of bonding environments within a symmetry group [29]. Fluxionality is analogous to a superposition of states for a chemical bonding system.

Overall, we observe increased C1s XAS signals in all ND samples in the 285–288.5 eV region, as some features are very distinct and close to the core-hole exciton at 289.0 eV (conduction band minimum, or CBM). ND-BBr_3_ has a linear increase in absorption in the 285–287.6 eV region due to newly created carbon states that would typically be assigned to π*(C=C) and π*(C=O) resonances, yet are more likely to be due to C-B-C bonds across the diamond surface akin to the CBC chain in B_4_C. At 288.4 eV, 0.6 eV below the CBM, there is a distinct peak assigned to the π*(C-B)_icosa_ state that has not been observed previously on diamond, yet is in excellent agreement with the main peak of B_4_C at the same energy. A similar transition in closo-carborane was attributed to the 10a’’(y) and 17a’(xz) states, which represent the carbon bound to 5 boron atoms in the crown of the icosahedron [62]. Our results suggest that hypervalent carbon bonding to boron is possible on diamond surfaces. ND-BCl_3_ has a unique 3-step rise in absorption with peaks at 286.4 eV, 287.5 eV, and 288.4 eV assigned to π* states. The 286.4 eV feature can be tentatively assigned to CBC-like chains across the diamond surface as denoted π*(CBC), while the 287.6 eV is assigned to the π*(C-B)_iso_ state as discussed above and consistent with conclusions reached by Caretti in studying their B*_x_*C_1-*x*_ thin films [56]. The ND-BH_3_ C1s spectra have signal issues in the 285 eV regime as mentioned previously, yet have features consistent with ND-BCl_3_ and ND-BBr_3_ with similar peaks at 286.4 eV, 287.5 eV, and 288.4 eV that are assigned to π* states. What is inconsistent for ND-BH_3_ is the lack of resonance in the sub-194 eV region during B1s measurements. ND-BH_3_ samples have the greatest decrease of C-O-H bonds based on DRIFTS data, and further investigations are needed to understand the role of reductive chemistry and boron templating when using BH_3_.

O1s XAS spectra revealed that acids on the ND surface are not affected by the boron chemistry, and new features arose in the shape resonance region indicative of oxygen-boron bond formation. Figure 6D contains a collection of control samples and boron-treated diamond samples, including ND-OH, with peaks at 530.4 eV and 536.5 eV assigned to π*(C=O) and σ*(C-O) transitions from carboxylic acids and alcohols. Boron-treated samples generally have the same π*(C=O) peak around 530.5 eV, while a shape resonance feature emerges at 538.1 eV that corresponds to the largest boric acid σ*(B-O) transition. Taking a differential spectrum of ND-BCl_3_ and ND-OH yields absorption increases at 527.8 eV, 530.1 eV, 538.9 eV, and 540.5 eV attributed to the new π*(B-O) and σ*(B-O) formed during the nucleophilic attack on the boron centers by alcohols. The spectral overlap of π*(B-O) and π*(C=O) resonances complicates the interpretation, and an examination of boric acid and ND-OH highlights the overlap. A significant decrease in absorption at 536.1 eV is consistent with the removal of σ*(C-O) transitions and supports a mechanistic picture that alcohols were converted to organoborates during the nucleophilic attack on the boron centers by alcohol groups on the diamond surface.

**Figure 7:**
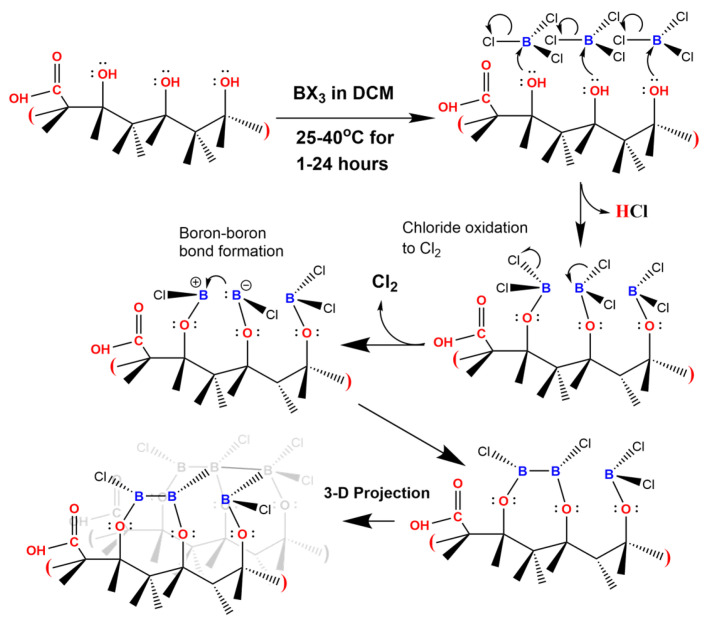
Schematic of the mechanism leading to the ultrathin boron layers on ND-OH surfaces. Tertiary alcohols on the diamond surface perform a nucleophilic attack on the trigonal boron centers, leading to the loss of chloride ions and protons. The number of HCl groups should be limited to the number of alcohols and trace water in the anhydrous DCM. The next step includes the loss of additional chlorides and the oxidative formation of Cl_2_. Boron-boron bond formation is achieved in plane with the diamond surface and is projected as a 3-D structure to show the layering that has been observed with SEM and other techniques.

**Mechanism for boron templating on HPHT ND surfaces.** The new chemistry and supporting spectroscopy with microscopy describe a unique path to covalently link boron to diamond surfaces, and we build a speculative mechanism to explain our results. The closet analogue to our work is the boron layering of diamond at temperatures of 1600–1700 K using powdered boron on diamond {111} surfaces [63] as the boron source and is in stark contrast from the mild conditions we employ at 298–312 K. The temperature difference highlights that selecting the correct boron precursors that are sufficiently electrophilic can accomplish sufficient reactivity. Figure 7 is a mechanism for the growth of ultra-thin boron layers on ND-OH that contains the loss of HCl and Cl_2_ in separate steps and the formation of boron-boron bonds across the diamond surface. Step #1 involves the alcohols performing a nucleophilic attack on the boron center to generate an alkoxyborochloride with the subsequent loss of a chloride ion and a proton to yield hydrochloric acid (recall the chemistry occurs in anhydrous DCM). Step #2 is similar to the generation of B_2_Cl_4_ from BCl_3_ covered by Holliday and Massey in 1962 and is likely to involve the oxidation of 2Cl^−^→Cl_2_ and reduction to generate boron-boron bonds [64,65]. In the electron pushing diagram, we depict chloride #1 as a leaving group while simultaneously showing chlorine #2 undergoing bond breaking and the sp^2^ hybridized electrons returning to the boron atom. Conformation of reddish Br_2_ and yellowish Cl_2_ solutions were performed with UV-visible spectroscopy and reinforce this crucial step. In step #3, the sp^2^ hybridized electrons on boron #2 initiate the boron-boron bond formation across the diamond surface. In order to depict the boron layering parallel to the surface, another diamond lattice is placed behind the original. Our spectroscopic data (DRIFTS, XPS, and XAS) supports a surface structure with both trigonal and tetrahedral boron bonding occurring simultaneously. The last panel depicts this heterogeneity. As a future direction, increasing boron-boron bond formation could be catalyzed by sodium napthalide (NaNaph) and is an area of investigation. Previously, boron nanoparticles were formed by reduction of BCl_3_ by NaNaph and appear to be robust [66]. Another avenue is to replicate the boron chemistry on a single crystal 111-terminated diamond and perform surface analysis and scanning probe microscopy to evaluate the boron layers. 111-terminated HPHT diamond macles from element 6 would be a natural substrate to conduct the comparative study.

## 4. Conclusions

This study establishes the first room temperature growth of ultrathin boron layers onto diamond surfaces by leveraging trigonal boron precursor reactivity with tertiary alcohols. A suite of techniques was used to fully probe the ND morphology, elemental composition, and surface structure. We discovered that the boron layers did not increase in thickness with extended reactions of up to 24 h, and the evidence suggests a self-terminating chemical mechanism similar to atomic layer deposition. Morphology of single ND and aggregated NDs was found, and etching may have played a role. Importantly, the bonding environments at the diamond surface show boron in trigonal and tetrahedral bonding geometries (i.e., ogano-borates), with boron-boron bonding also occurring. XAS data supports both boron-boron and diamond-boron bond formation, with a significant increase in mid-gap states in the 285–288.3 eV region. Direct diamond-boron bonds may occur through reactions of reconstructed sp^2^ groups with the boron precursors, similar to small-molecule reactions with alkenes. This report highlights that room-temperature chemistry under air-free conditions can generate covalently bound boron layers on ND surfaces when the proper electrophiles are used. Researchers in materials science, biolabeling, and quantum sensing will find the results useful for designing new layered structures, boron neutron capture therapy applications, and manipulating the surface dipole moment of diamond for near-surface NV center-based detection.

## 5. Patents

Information from this study was used to submit US Patent App. 18/454,139 and is entitled “Boronated nanoscale substrate and uses thereof.”

## Figures and Tables

**Table 1:** Inelastic mean free paths (IMPF) of C1s, B1s, and O1s electrons based on the TPP-2M equation (Tanuma-Powell-Penn) for diamond, B_4_C, and SiO_2_ with sensitivity factors based on transmission functions in VAMAS files. IMPF values were calculated with E = 1486.7 eV for an Al Kα source.

Element	Edge	IMPF (nm)	Sensitivity Factors
Carbon (Diamond)	1s	3.25	1.0
Boron (Boron Carbide)	1s	3.01	0.486
Oxygen (SiO_2_)	1s	2.81	2.93

**Table 2:** Quantitative summary of XPS Survey Scans using VAMAS file types for % atomic concentrations analysis (10% error assumed for all values).

Sample	Surface Termination	Carbon %	Boron %	Oxygen %	Halogen %
ND-OH-1	Alcohols	86.9	0.0	13.1	0.0
Boron	Boron Oxide	15.3	57.0	27.7	0.0
Boron Carbide	Boron Oxide	52.1	30.0	17.9	0.0
ND-BBr_3_-1	B-B, B-C, B-O	89.7	3.1	6.5	0.7
ND-BBr_3_-2 + 1 month	B-B, B-C, B-O	69.4	2.5	27.7	0.4
ND-BBr_3_-3	B-B, B-C, B-O	82.2	3.4	14.2	0.2
ND-BCl_3_-1	B-B, B-C, B-O	76.6	2.8	20.4	0.2
ND-BCl_3_-2 + 1 month	B-B, B-C, B-O	90.0	0.6	9.0	0.4
ND-BCl_3_-3	B-B, B-C, B-O	83.8	1.7	14.1	0.4
ND-BH_3_-1	B-B, B-C, B-O	78.5	2.0	19.5	0.0
ND-BH_3_-2 + water	B-B, B-C, B-O	82.0	0.8	17.2	0.0
ND-BH_3_-3	B-B, B-C, B-O	82.7	1.6	15.8	0.0

## Data Availability

Original datasets will be provided upon request and can be submitted to Abraham Wolcott.

## Supplementary Materials

The following supporting information can be downloaded at: https://www.mdpi.com/article/10.3390/nano14151274/s1, and includes SEM and EDS of oxidized NDs, B_4_C and elemental boron, *H*RTEM, EDS, and EELS of NDs treated with BBr_3_, and a solubility study of ND-BCl_3_ in various solvents with dynamic light scattering (DLS). References [67,68,69,70,71,72,73,74,75,76,77,78,79] are cited in the supplementary materials.
