# Supplementary material for: Ultrathin Boron Growth onto Nanodiamond Surfaces via Electrophilic Boron Precursors
Source: Nanomaterials (Basel). 2024 Jul 29;14(15):1274. doi: 10.3390/nano14151274 (PMC11314373; doi:10.3390/nano14151274)
Supplement: Supplementary file 1 [file nanomaterials-14-01274-s001.zip › nanomaterials-3063623-supplementary.pdf]

Supporting Online Material

Ultrathin Boron Growth onto Nanodiamond Surface via Electrophilic Boron Precursors

Krishna Govindaraju [1,†], Tyanna Supreme [1,†], Daniel N. Labunsky [1,†], Nicole Martin [1], Juan Miguel Del Rosario [1], Alana Washington [1], Ezhioghode O. Uwadiale [1], Solomon Adjei II [1], Sandra Ladjadj [1], Cynthia V. Melendrez [1,2], Sang-Jun Lee [3], Maria V. Altoe [4], Avery Green [5], Sebastian Riano [5], Sami Sainio [3,6] and Dennis Nordlund [3] and Abraham Wolcott [1,*]

1 Department of Chemistry, San José State University, 1 Washington Square, San José, CA 95192, USA; krishna.govindaraju@sjsu.edu (K.G.); tyanna.supreme@sjsu.edu (T.S.); daniel.labunsky@sjsu.edu (D.N.L.); nicole.martin@sjsu.edu (N.M.); juanmiguel.delrosario@sjsu.edu (J.M.D.R.); alana.washington@sjsu.edu (A.W.); ezhioghode.uwadiale@sjsu.edu (E.O.U.); solomon.adjei@sjsu.edu (S.A.II); sandra.ladjadj@sjsu.edu (S.L.); cymel123@slac.stanford.edu (C.V.M.)

2 Linac Coherent Light Source, SLAC National Accelerator Laboratory, 2575 Sandhill Road, Menlo Park, CA 94025, USA

3 Stanford Synchrotron Radiation Lightsource, SLAC National Accelerator Laboratory, 2575 Sandhill Road, Menlo Park, CA 94025, USA; sangjun2@slac.stanford.edu (S.-J.L.); sami.sainio@carbon.fi (S.S.); nordlund@slac.stanford.edu (D.N.)

4 The Molecular Foundry, Lawrence Berkeley National Laboratory, 1 Cyclotron Road, Berkeley, CA 94720, USA; mvpaltoe@lbl.gov

5 Covalent Metrology, 927 Thompson Pl, Sunnyvale, CA 94085, USA; avery@covalentmetrology.com (A.G.); sebastian1430@gmail.com (S.R.)

6 Microelectronics Research Unit, University of Oulu, Pentti Kaiteran Katu 1, Linnanmaa, P.O. Box 4500, 90014 Oulu, Finland

* Correspondence: abraham.wolcott@sjsu.edu

† These authors contributed equally to this work.

Table of Contents

Section 1. HRTEM, EDS and EELS of NDs treated with BBr_3. *page S3-5*

Section 2. SEM and EDS of ND-OH, B_4C and Elemental Boron. *page S6-9*

Section 3. **Solubility study of ND-BCl_3 in various solvents with Dyanmic Light Scattering (DLS).** *page S10-12*

Section 4. XPS Difference Spectra of Water Dispersed ND-BBr_3 samples. *page S13*

Section 5. **References** *page S14*

Section 6. **Russian and English Version of Gavrilin et al article** *page S15-21*

Section 1. **HRTEM, EDS and EELS of NDs treated with BBr_3**. An example of the ND-BBr_3 samples shows a drastically altered morphology as observed by electron microscopy in S1A. The ND-BBr_3 samples are rounded in comparison to the shard-like starting material of HPHT NDs (see S3), NDs are crosslinked or aggregated and are thinly coated with boron. When viewing the HRTEM image of S1A there is difficulty in distinguishing individual particles and is likely due to the similar transmission and scattering cross sections of carbon (Z=6) and boron (Z=5) atoms by the electron beam.(*1*) In contrast, a silica (SiO_2; Z of Si=14) coated HPHT ND has pronounced differences in contrast and silica thickness measurements are straightfoward.(*2-7*) The elemental composition in a point scan can result in a spectrum similar to S1B, whereby the dominant C Kα peak hinders a measurable B Kα peak at the concentrations seen in this work. Scanning transmission electron microscopy energy dispersive X-ray spectroscopy or STEM-EDS was used to provide nanometer spatial resolution of the ND-BBr_3 cluster and produced maps in S1C-E. Because B Kα and C Kα emission peaks occur at 183 eV and 277 eV, respectively, overlap in counts can be incorrectly interpreted as B Kα emission.(*8*) Spatially, the B Kα counts are similar in position due to the underlying diamond with a strong C Kα intensity. O Kα counts being energetically separated at 525 eV have no overlap and is presentative of the oxygen composition of the ND-BBr_3 sample. Notably, post reaction, the presence of bromine can also be clearly seen with Br Lα emission at 1480 eV and reinforces that trace Br still resides on the ND-BBr_3 sample, yet the bonding environment is not wholly understood.

The presence of diamond within the ND-B clusters is definitively confirmed with the use of electron energy loss spectroscopy or EELS. EELS is based on the interaction of the incident electron beam within the TEM microscope and the inelastic collisions with a sample.(*9*) EELS can provide information about chemical composition and electronic structure in much the same way as XAS. EELS data in S2 shows much of the electronic structure of diamond with the 2nd absolute bandgap "dip" at 302 eV. The EELS spectrum cannot be confused with the ultrathin carbon film used in Ted Pella grids (#01824). Unlike XAS measurements, the diamond core-hole exciton at 289.0 eV is not observed and is due to the lack of energy resolution in the microscope.(*10-12*) The TEM grid shows a π*(C=C) peak due to sp^2 bonding environments within the carbon film and then broad σ* shape resonances above 290 eV.

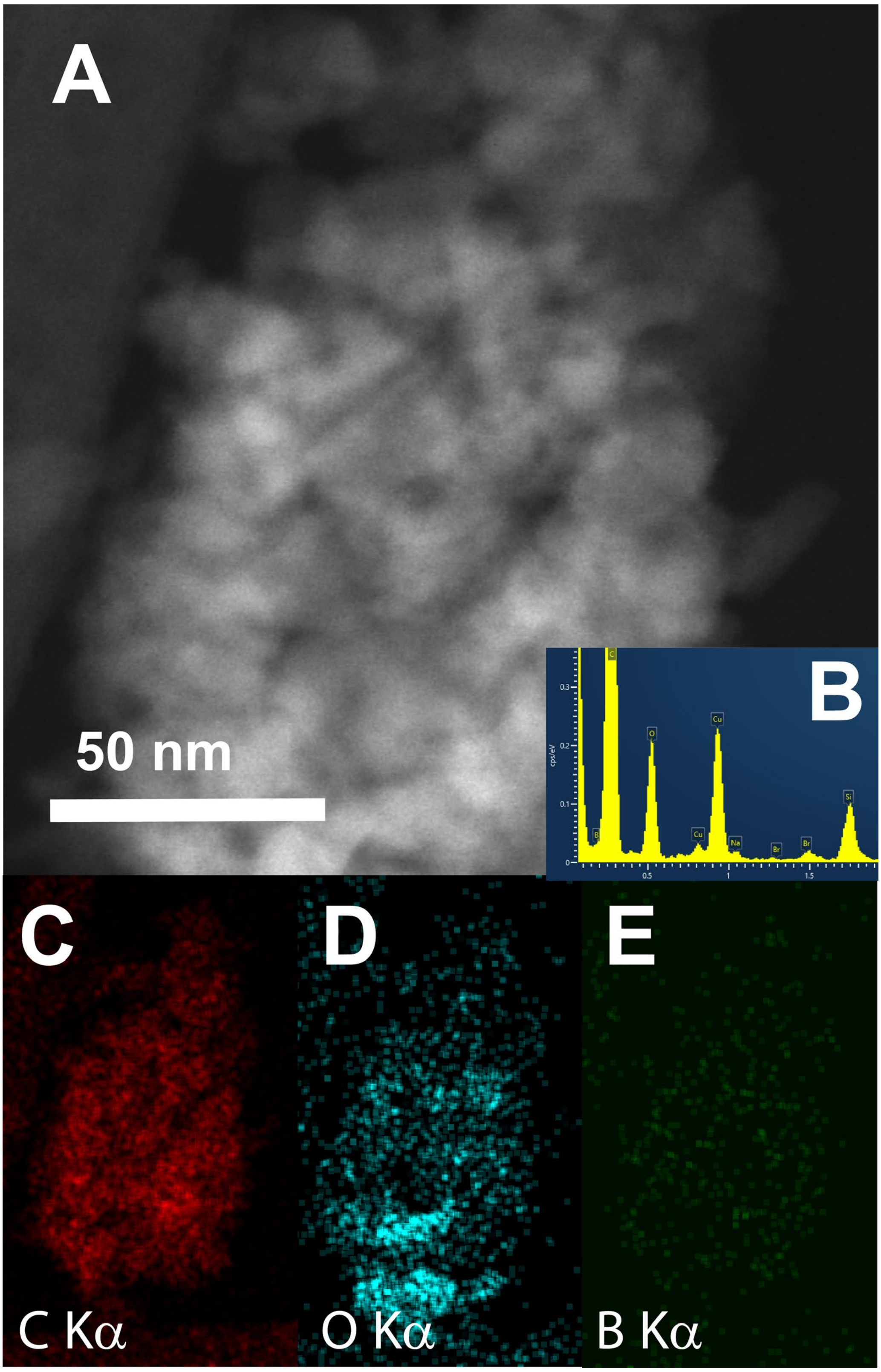

S1. A cluster of NDs is seen with HRTEM, STEM-EDS and EDS spectrum and shows the difficulty in resolving the individual NDs reacted with boron precursors and the boron signals from EDS. The X-ray emission lines from C Kα overlap with B Kα creating ambiguous results and requiring further characterization with XPS and XAS.

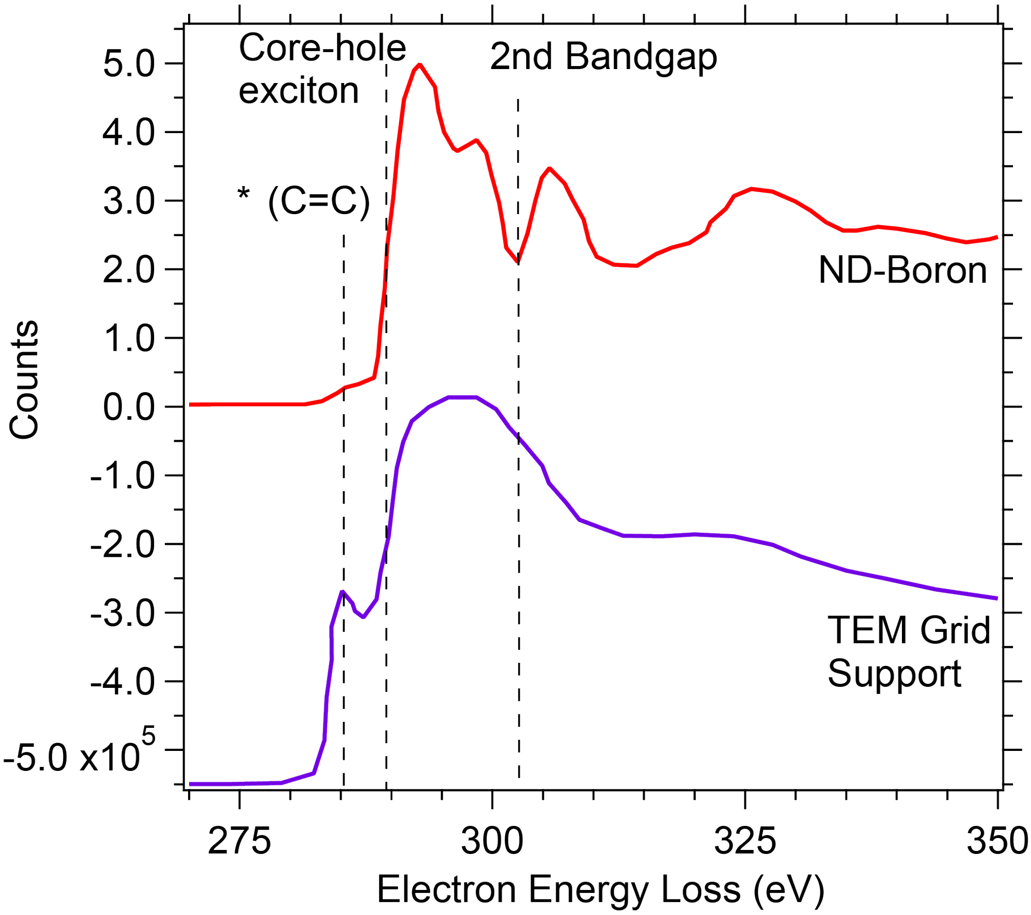

S2. Electron energy loss spectroscopy of the boron treated NDs reveals the standard electronic structure of the diamond including the distinct dip of the 2nd bandgap present at 302 eV. The EELS measurements was not able to resolve the sharp diamond core-hole exciton. The holey carbon TEM grid also has an amorphous carbon signal with a sharp π*(C=C) feature at ~285 eV.

Section 2. SEM and EDS of ND-OH, B_4C and Elemental Boron.

To verify the morphology and size of ND-OH samples before and after reactions with trigonal boron precursors, traditional electron microscopy (high resolution SEM and high resolution TEM) and EDS. Microscopy and chemical analysis of B_4C and elemental boron powders was carried out by staff scientists at Covalent Metology in Santa Clara, CA. Typically, EDS is very effective in generating elemental maps of layered and core-shell structures. This section highlights the inherent difficulty in qualitatively and quantitatively characterizing thin layers of boron on a diamond substrate.

ND-OH SEM and EDS. First, the aerobically oxidized HPHT ND samples were examined as a baseline metric of the sample morphology, EDS carbon and oxygen signals and background signals. As seen in figure S3, the nanoscale morphology of the HPHT NDs can be clearly seen with a jagged and irregular morphology and a size distribution of 10-100 nm.(*12*, *13*) ND-OH colloids were deposited on silicon wafers and EDS data was collected as seen in S4. Carbon, oxygen and silicon signals were quantified as 81%, 3.2% and 15.8%, respectively. EDS is an electron-in and X-ray-out

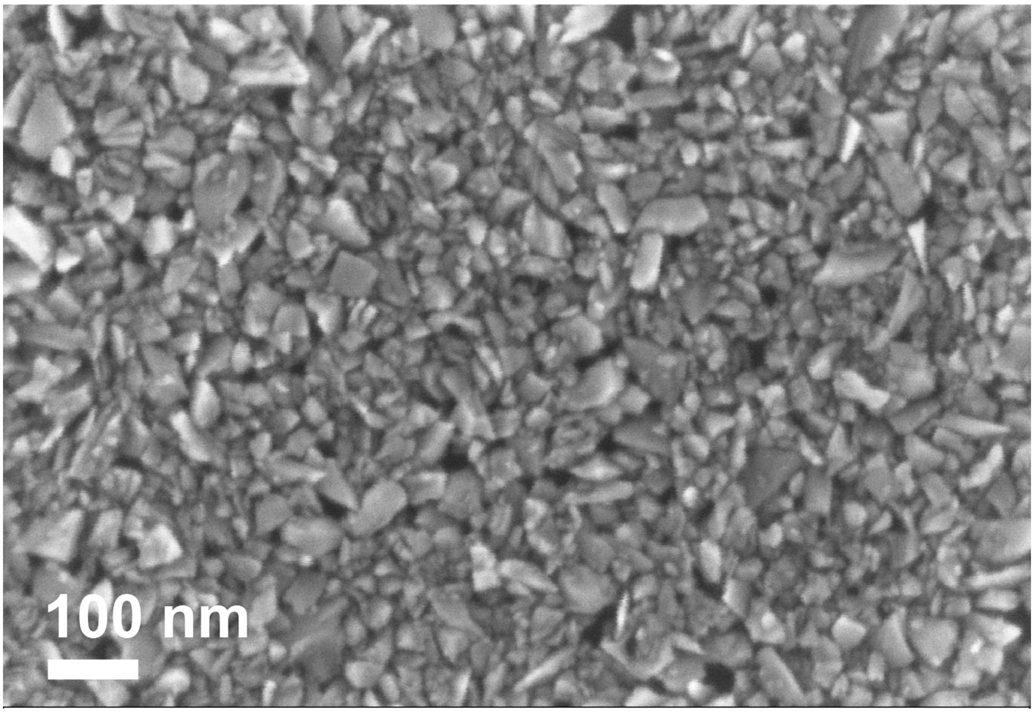

S3. High resolution SEM of an oxidized HPHT ND-OH sample. ND-OH samples from microdiamant range in size from 10-100 nm, have a platelet structure and are iregular shaped. Cleavage of macroscopic HPHT diamond particles during ball millling generates the morphology pattern.

technique and provides elemental information throughout the entirety of the sample.

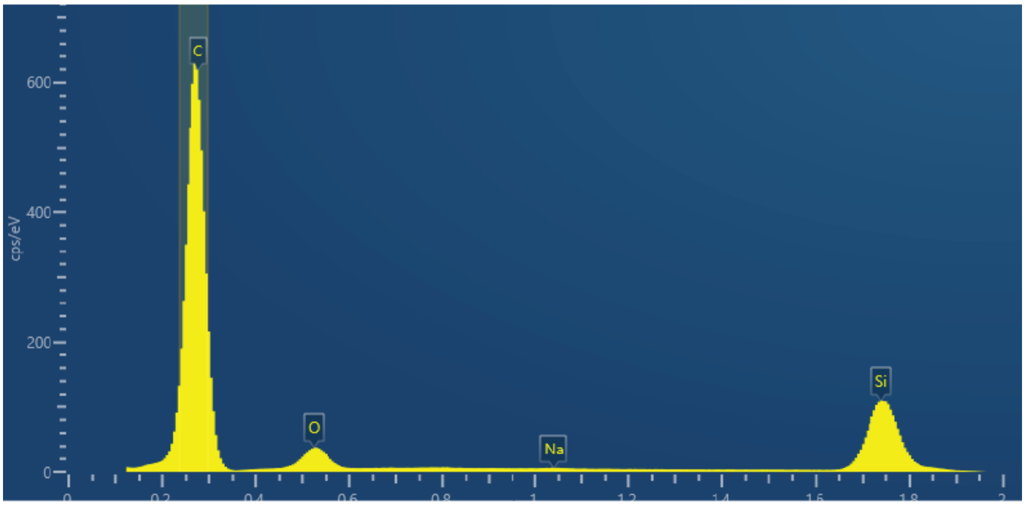

S4. EDS spectrum from ND-OH samples deposited on a silicon wafer. Carbon, oxygen and Si atoomic percentages were 81%, 3.2% and 15.8%, respectively.

Control Boron Carbide (B_4C) SEM and EDS. B_4C powders were acquired from Ted Pella Inc. and deposited onto silicon wafers and imaged via SEM and showed single crystal sizes ranging from ~30-100 μm. The powders have an irregular morphology with sharp edges and facets that can be clearly seen as the magnification is increased in S5. As the accelerating voltage was increased the calculated atomic percentages were found to vary with the carbon signals increasing. Accelerating voltages from 2→10 keV resulted in B Kα, C Kα and O Kα signals of 71.4%, 27.2 and 1.4% changing to 55.9%, 43.0% and 1.1%, respectively. A stoichiometry of 4:1 for the B_4C was not observed, yet illustrates the proximity of the B Kα and C Kα emission peaks and the difficulty in getting accurate quantification even in a known sample.

Elemental boron powder (B) SEM and EDS. High purity B powders were acquired from Sigma Aldrich and imaged via SEM and showed single crystal sizes ranging from ~500 nm up to 10 μm. Boron is a reddish brown powder and has a rounded morphology and are seen isolated and aggregated in S6. Accelerating voltages of 4 keV and 5 keV were used and showed a boron rich sample with small concentrations of carbon and oxygen. See the table inset within S6 for atomic % concentrations. The C Kα emission peak is still discernible in the EDS spectrum and is due to adventitious carbon. O Kα features are due to oxidation of the boron powders in atmospheric conditions and adsorbed water.

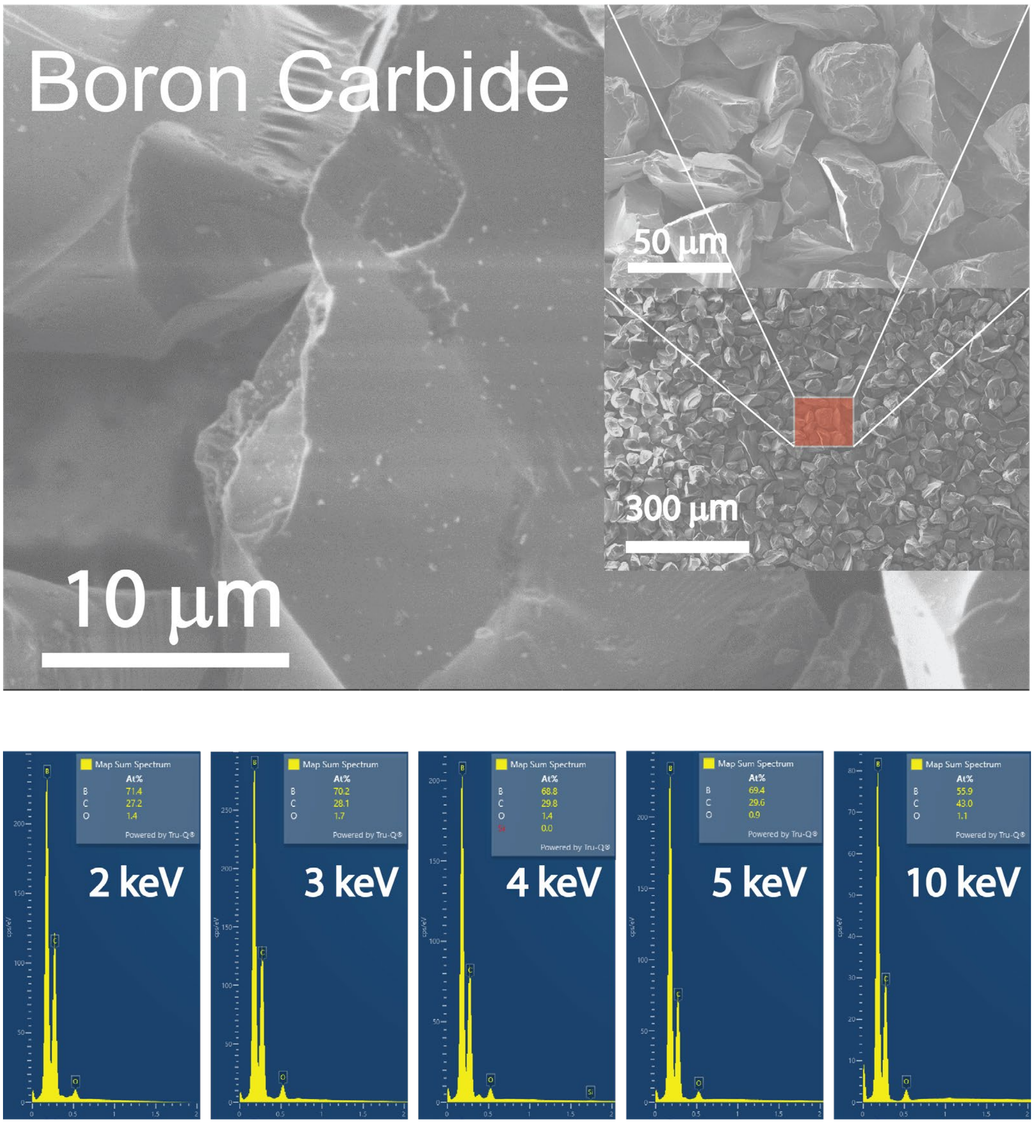

S5. SEM images of powdered boron carbide at varying magnification reveals the size and morphology of the control samples. Typical particle diameters were from 30-100 µm and had sharp edges and facets that are highlighted in the highest magnification image. EDS data at varying accelerating voltages revealed the changing atomic percentages of the B4C sample based on EDS counts. Decreasing the accelerating voltage from 10→2 keV increased the detectable B Kα X-rays and resulted in atomic percentages approaching the B_4C stoichiometry.

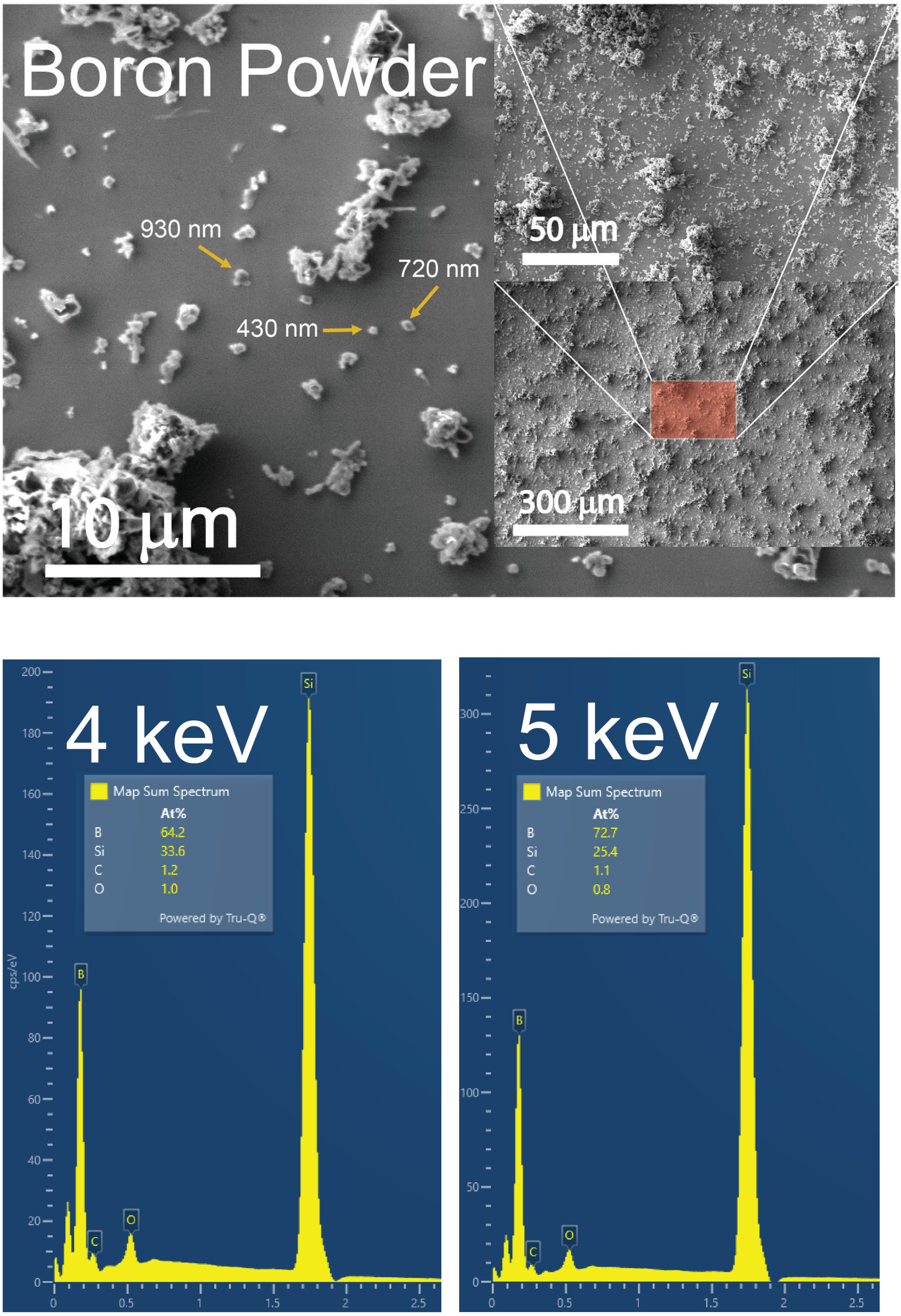

S6. SEM images of powdered boron at varying magnification reveals the size and morphology of the control samples along with its elemental composition. Individual particles range in size and clusters of 430 nm, 720 nm and 930 nm are highlighted. EDS scans at 4 keV and 5 keV show the changes in quantitative values of B, C and O. Trace contributions of C and O are quantified at approximately 1% for both accelerating voltages. Note the close proximity of the B Kα and C Kα peaks in energy.

Section 3. Solubility study of ND-BCl_3 in various solvents with Dynamic Light Scattering (DLS). Reaction of the alcohol-terminated ND-OH samples dispersed in DCM drastically alters their solubility and floculation occurs. The electron deficient nature of the boron overlayers on nanoscale diamond in solution are wholly unknown and required investigation. 4 non-polar solvents were chosen in parallel with water. Table 1 contains the dipole moments of each solvent along with the diameters of the ND-BCl_3 particles based on DLS histograms. In a study by Pickering, boron nanoparticles were synthesized in glyme with BBr_3 and later solubilized in hexane after a ligand exchange reaction with octanol.(*14*) Interestingly, the boron-bromide bonds were reactive to octanol via the alcohol groups to yield an octoxy-capped boron nanoparticle and NMR was used for characterization. NMR is not sensitive enough to detect the trace concentrations of boron in the ND-B system.

The diameter of ND-BCl_3 in octane, toluene, octadecene, chlorobenzene and water shows a heterogenous distribution ranging from 100-1300 nm. Gaussian peak fitting was applied to the DLS histograms and are seen in S7 and summarized in table 1. The table outlines the dipole moment of each solvent given in Debye(*15*), the Gaussian peak fit diameter in nanometers (nm) and the distribution being monomodal (mono), bimodal (bi) or trimodal (tri). Starting with octane, having a dipole moment of 0.00 D, the size distribution has three peaks at 155 nm, 330 nm and 540 nm, while toluene with a 0.31 D moment has a monomodal distribution centered at 310 nm. Now compare these values to ND-OH in water with an average diameter of ~40 nm as seen in the red trace. Cross linking or aggregation should be contrasted with the starting material of 40 nm and therefore a 155 nm or 310 nm diameter can be interpreted as having 4 or 8 crosslinked nanoparticles, respectively. Next, 1-Octadecene with a 0.38 D moment, is a 18 carbon non-polar solvent with a single double bond and commonly used in II-VI and IV-VI nanoparticle synthesis.(*16, 17*) ND-BCl_3 dispersed in octadecene had 2 peaks at 400 nm and 770 nm showing a higher level of aggregation than both octane and toluene. Chlorobenzene, with an increased dipole of 1.54 D, aided solubility and produced a bimodal distribution with peak positions at 190 nm and 300 nm. Chlorobenzene and toluene differ by the substitution of a -chloride and methyl group, respectively, of the benzene ring while providing a comparison of the delocalized pi-bonding within the benzene ring and the electron withdrawing capabilities of the chloride. Water, the most polar and only reactive solvent to disperse the ND-BCl_3 samples showed a bimodal distribution of 130 nm and 770 nm. This size represents both the smallest and largest size distributions within the solubility study. We know that boron was removed from the ND surface based on XPS and was likely in the form of boric acid during dissolution. The dissolving of boric acid may have led to delinking of NDs encased in the ultrathin boron

shells. A 130 nm particle size indicates approximately 2-3 NDs are involved in a cluster. Delinking may also have allowed for aggregation to occur as evidenced by the 2nd peak centered at 770 nm with a distribution from 500→1300 nm. These results suggest a mechanism in which ND-BCl_3 particles may delink and reassemble as aggregates at varying rates in the aqueous solution.

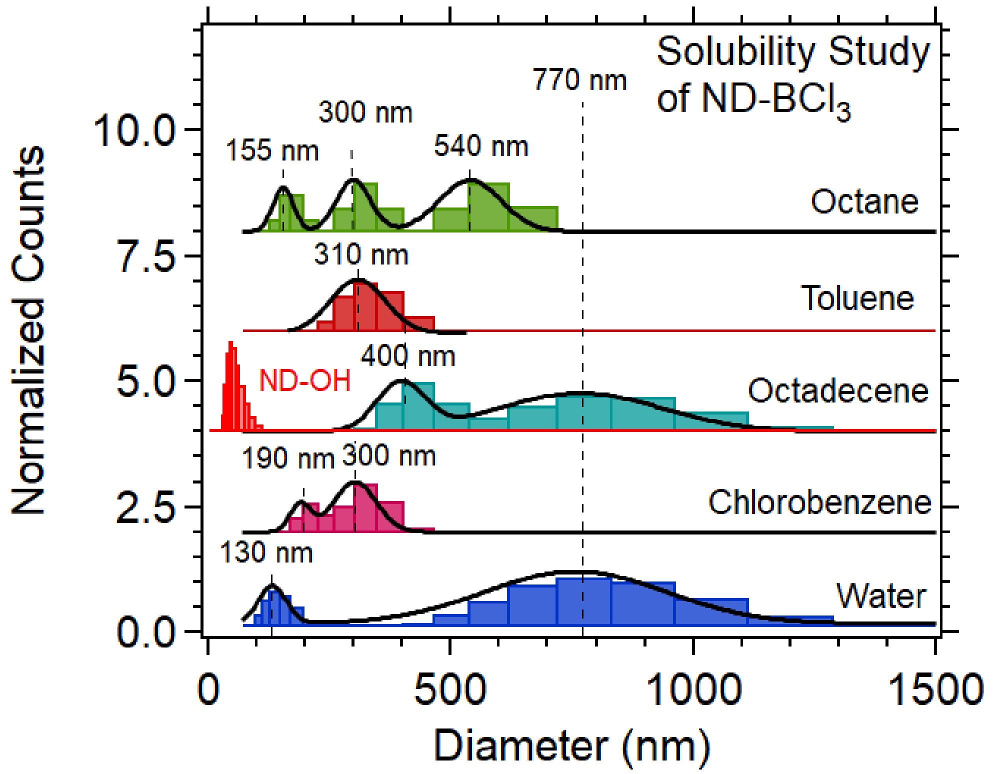

S7. DLS solubility study of ND-OH samples dispersed in DCM and reacted with BCl_3. After the boron treatment the samples were not well solubilized in DCM and this study was initiated. Non polar solvents and water were used to investigate the colloidal stability of the ND-B constructs. ND-OH in red is the starting material dispersed in water with a mean diameter of ~40 nm.

Table 1: Dipole moments and average diameters of ND-BCl_3 in various solvents

Solvent	Dipole moment (D)	Diameter #1 (nm)	Diameter #2/3 (nm)	Size Dist.
**Octane**	0.00	155	330/540	Tri
**Toluene**	0.31	310	--	Mono
**Octadecene**	0.38	400	770	Bi
**Chlorobenzene**	1.54	190	300	Bi
**Water**	1.87	130	770	Bi
**ND-OH in water***	1.87	40	--	Mono
**ND-BCl_3 in DCM****	1.14	--	--	----

*=data shown **= data not shown

Section 4. XPS Difference Spectra of Water Dispersed ND-BBr_3 samples. Linear background subtractions of ND-BBr_3 and ND-BBr_3 + H_2O are below in concert with the difference spectrum. While the boron % atomic concentration was reduced by 23% after dispersion in water, the remaining boron bonding environment is largely due to $(B\text{-}O)^{+3}$ as expected. There were increases in B1s counts at 185.2 eV and 190.7 eV and decreases in intensity at 181.8 eV, 183.2 eV and 188.6 eV. One would expect elimination of the Br3p spin-orbit peaks due to water exposure and the generation of HBr. Unexpectedly, the B1s intensity in the range of 182-185 eV remains and its chemical signature is worthy of further investigation.

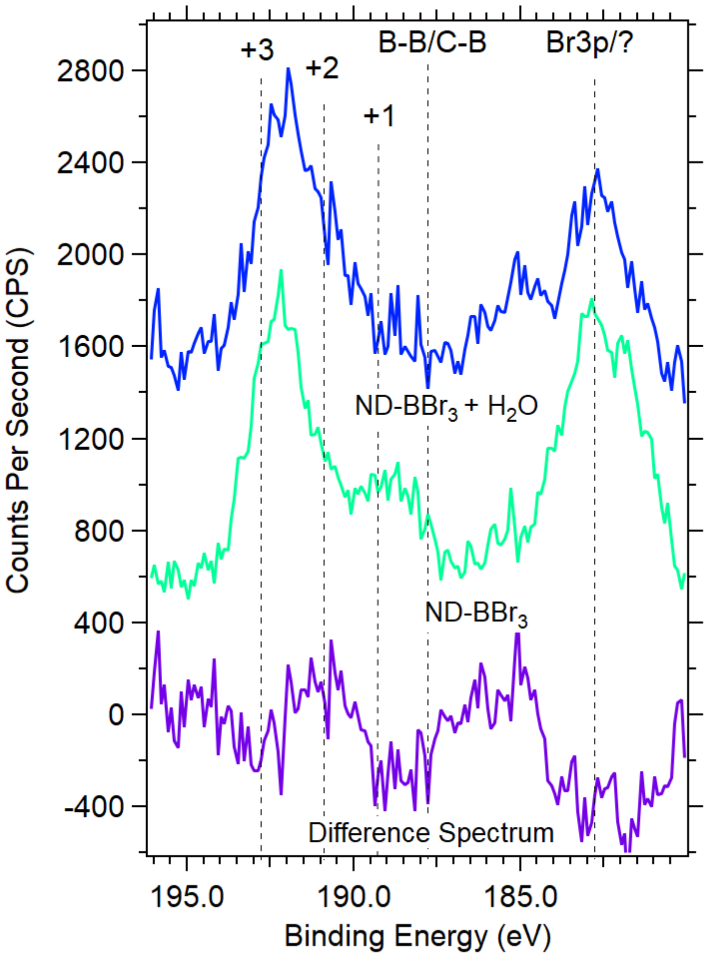

S8. XPS of ND-BBr_3 before and after being dispersed in water with sonication as a colloid. Water exposure reduced the quantitative amount of boron by 23%, yet boron remained on the surface covalently bound to diamond. B1s peaks in the range of 182-184 eV should have been eliminated if they were due to Br3p and may suggest that B1s binding energy is reduced due to electron donation.

References

1. L. E. Franken, K. Grünewald, E. J. Boekema, M. C. A. Stuart, A Technical Introduction to Transmission Electron Microscopy for Soft-Matter: Imaging, Possibilities, Choices, and Technical Developments. *Small* **16**, 1906198 (2020).
2. T. Rendler *et al.*, Optical imaging of localized chemical events using programmable diamond quantum nanosensors. *Nature Communications* **8**, (2017).
3. J. Neburkova, J. Vavra, P. Cigler, Coating nanodiamonds with biocompatible shells for applications in biology and medicine. *Current Opinion in Solid State & Materials Science* **21**, 43-53 (2017).
4. I. Rehor *et al.*, in *Carbon Nanomaterials for Biomedical Applications,* M. Zhang, R. R. Naik, L. Dai, Eds. (2016), vol. 5, pp. 319-361.
5. V. Petrakova *et al.*, Imaging of transfection and intracellular release of intact, non-labeled DNA using fluorescent nanodiamonds. *Nanoscale* **8**, 12002-12012 (2016).
6. I. Rehor *et al.*, Fluorescent Nanodiamonds Embedded in Biocompatible Translucent Shells. *Small* **10**, 1106-1115 (2014).
7. P. J. Sandoval *et al.*, Quantum Diamonds at the Beach: Chemical Insights into Silica Growth on Nanoscale Diamond using Multimodal Characterization and Simulation. *ACS Nanoscience Au*, (2023).
8. M. Scimeca, S. Bischetti, H. K. Lamsira, R. Bonfiglio, E. Bonanno, Energy Dispersive X-ray (EDX) microanalysis: A powerful tool in biomedical research and diagnosis. *Eur J Histochem* **62**, 2841 (2018).
9. R. F. Egerton, Electron energy-loss spectroscopy in the TEM. *Reports on Progress in Physics* **72**, 016502 (2009).
10. A. Stacey, B. C. C. Cowie, J. Orwa, S. Prawer, A. Hoffman, Diamond C 1s core-level excitons: Surface sensitivity. *Physical Review B* **82**, (2010).
11. J. F. Morar, F. J. Himpsel, G. Hollinger, G. Hughes, J. L. Jordan, OBSERVATION OF A C-1S CORE EXCITON IN DIAMOND. *Physical Review Letters* **54**, 1960-1963 (1985).
12. A. Wolcott *et al.*, Surface Structure of Aerobically Oxidized Diamond Nanocrystals. *Journal of Physical Chemistry C* **118**, 26695-26702 (2014).
13. D. R. C. Uri Zvi, Adam Weiss, Aidan R. Jones, Lingjie Chen, Iryna Golovina, Xiaofei Yu, Stella Wang, Dmitri V. Talapin, Michael E. Flatté, Aaron P. Esser-Kahn, Peter C. Maurer, Engineering Spin Coherence in Core-Shell Diamond Nanocrystals. *arXiv*, (2023).
14. A. L. Pickering, C. Mitterbauer, N. D. Browning, S. M. Kauzlarich, P. P. Power, Room temperature synthesis of surface-functionalised boron nanoparticles. *Chemical Communications*, 580-582 (2007).
15. W. M. Haynes, *CRC Handbook of Chemistry and Physics (97th ed.).* (CRC Press. , 2016).
16. N. C. Anderson, M. P. Hendricks, J. J. Choi, J. S. Owen, Ligand Exchange and the Stoichiometry of Metal Chalcogenide Nanocrystals: Spectroscopic Observation of Facile Metal-Carboxylate Displacement and Binding. *Journal of the American Chemical Society* **135**, 18536-18548 (2013).
17. A. Wolcott *et al.*, Anomalously Large Polarization Effect Responsible for Excitonic Red Shifts in PbSe Quantum Dot Solids. *Journal of Physical Chemistry Letters* **2**, 795-800 (2011).

Gavrilin, A.V., Luk'yanov, I.M., Smirnov, E.P., & Aleskovskij, V.B. (1987).

Interaction of boron halides with carbon preparations.

Zhurnal Obshchej Khimii, 57(6), 1350-1352.

(J. Gen. Chem. USRR, English translation)

U.S. Department of Energy

Office of Scientific and Technical Information (OSTI):

https://www.osti.gov/biblio/6961962

International Nuclear Information System (INIS) repository:

https://inis.iaea.org/search/searchsinglerecord.aspx?recordsFor=SingleRecord&RN=190 16605

We are grateful to the current ZHURNAL OBSHCHEI KHIMII / Russian Journal of General Chemistry editorial board, who has generously provided us with the full text of this article and has explicitly granted permission for its diffusion.

We thank Drs. Krivtsov and Ilkaeva, assistant professor and researcher, respectively, in the Department of Chemical and Environmental Engineering in the University of Oviedo for their disinterested assistance in translating the text to English.

[6] *Lucken E. A. C.* Nuclear Quadrupole Coupling Constants. — New York, London: Acad. Press, 1969. 360 p.
[7] *Фешин В. П., Воронков М. Г., Романенко Л. С., Игнатьева Л. П., Долгушин Г. В.* — ЖОХ, 1984, т. 54, вып. 6, с. 1312—1314.
[8] *Гущин С. М., Петухов С. А., Брюхова Е. В., Семин Г. К.* — Изв. АН СССР. Сер. хим., 1983, № 8, с. 1920—1922.
[9] *Фешин В. П., Сапожников Ю. Е., Долгушин Г. В., Ясман Я. Б., Воронков М. Г.* — Докл. АН СССР, 1979, т. 247, № 1, с. 158—160.
[10] *Семин Г. К., Бабушкина Т. А., Якобсон Г. Г.* Применение ядерного квадрупольного резонанса в химии. — М.: Химия, 1972. 536 с.
[11] *Фешин В. П., Воронков М. Г.* — Докл. АН СССР, 1977, т. 236, № 5, с. 1166—1169.
[12] *Jung M. E., Mazurek M. A.* — Synthesis, 1978, p. 588.
[13] *Воронков М. Г., Комаров В. Г., Албанов А. И., Коротаева И. М., Дубинская Э. И.* — Изв. АН СССР. Сер. хим., 1981, № 6, с. 1391—1392.
[14] *Воронков М. Г., Комаров В. Г., Албанов А. И., Дубинская Э. И.* — Изв. АН СССР. Сер. хим., 1978, № 11, с. 2623—2624.

Институт органической химии
Сибирского отделения
Академии наук СССР
Иркутск

Поступило в Редакцию
5 мая 1986 г.

УДК 541.183.5 : 546.27 *Журнал общей химии, 1987, т. 57, вып. 6*

ВЗАИМОДЕЙСТВИЕ ГАЛОГЕНИДОВ БОРА С ПРЕПАРАТАМИ УГЛЕРОДА

А. В. Гаврилин, И. М. Лукьянов, Е. П. Смирнов, В. Б. Алесковский

Рассмотрение твердых углеродных веществ (алмаз, графит) с позиций остовной гипотезы [1] позволило выявить химическое строение макрорадикала и функциональных групп, а также решить ряд задач по направленному синтезу химически монослойных элементгалогенидных и элементоксидных структур [2]. Публикации о взаимодействии галогенидов бора с углеродными материалами немногочисленны и в основном посвящены интеркалированным соединениям графита [3], хотя не меньший научный и практический интерес представляет синтез и использование боруглеродных соединений на основе неграфитовых углеродных материалов, особенно в связи с задачами получения полупроводниковых алмазов и каталитической графитации переходных форм углерода. В настоящей работе проведено исследование взаимодействия паров трихлорида и трибромида бора с оксифункциональными группами поверхности алмаза и технического углерода при давлении паров в диапазоне 0.33—3.52 кПа и в интервале температур 32—200 °С.

На рис. 1 приведены типичные кинетические изотермы взаимодействия углеродных препаратов с парами галогенидов. В опытах с BCl_3 после откачки адсорбата десорбция не наблюдалась, в то время как в опытах с BBr_3 десорбция была весьма существенна. С увеличением температуры опыта (рис. 1, *а*) количество способного к десорбции продукта падает, что характерно для физического взаимодействия, и при температуре опыта 200 °С десорбции практически нет. Количество необратимо адсорбированного BBr_3 практически постоянно в исследованном температурном интервале (рис. 1, *а*). Полученные кинетические кривые хорошо описываются уравнением

$$(1-\theta)=\exp(-kt),$$

где θ — степень заполнения поверхности к моменту времени t, $k=$ $=k_0 \exp(-E_a/RT)$ — константа скорости, E_a — энергия активации, R — универсальная газовая постоянная, T — абсолютная температура. При расчетах принимали, что $\theta=1$ при величинах адсорбции 4.9, 15 и

42 мг/г для систем BCl_3—алмаз, BBr_3 — алмаз и BBr_3 — технический углерод соответственно. При кинетических расчетах для систем BBr_3 — алмаз и BBr_3—технический углерод учитывали только необратимую адсорбцию, для чего проводили промежуточную десорбцию (рис. 1, *а*) и находили величину необратимой адсорбции в данный момент времени.

Для всех исследованных систем скорость хемосорбции описывается уравнением для однородной поверхности:

$$W=\left(\frac{\partial(1-\theta)}{\partial t}\right)_{p=\text{const}}=-k\exp(-kt).$$

Величины энергий активации и предэкспоненциальных множителей в уравнении константы скорости, определенные из зависимостей $\ln k = f(1/T)$, составили: 18 ± 4 кДж/моль и 21 ± 5 с$^{-1}$ для системы BCl_3—алмаз и 11 ± 4 кДж/моль и 1.1 ± 0.4 с$^{-1}$ для системы BBr_3—алмаз.

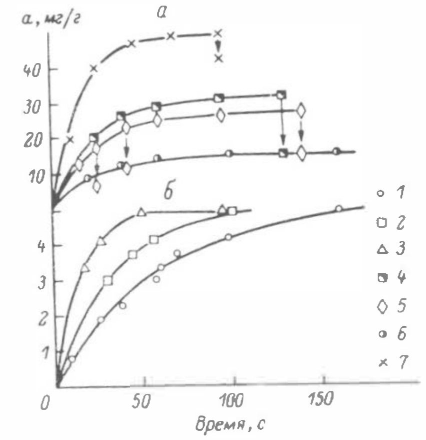

Рис. 1

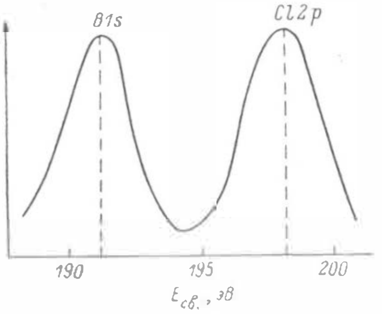

Рис. 2

Рис. 1. Кинетические изотермы взаимодействия бромида бора (*а*) и хлорида бора (*б*) с алмазом (*1—6*) и с техническим углеродом (*7*) при температуре, °С: *1* — 32; *2* — 58; *3*, *4* — 100; *5* — 150; *6*, *7* — 200. *1—3* — 0.53, *4—6* — 3, *7* — 1.5 кПа. *Стрелками* показана десорбция.

Рис. 2. B1*s* и Cl2*p* — линии рентгеноэлектронного спектра алмаза после взаимодействия с хлоридом бора.

Взаимодействие галогенидов бора с оксигруппами углеродных препаратов можно описать схемой:

$$\{\dot{C}\}_m(OH)_n + xBHlg_3 \longrightarrow \{C\}_m(OH)_{n-x-r}(OBHlg_2)_{x-r}(O_2BHlg)_r + (x+r)HHlg,$$

где $\{C\}_m$ — углеродный макрорадикал.

Сопоставление адсорбционных измерений с данными химического анализа для системы хлорид бора—алмаз

Температура опыта, °С	Поверхностная концентрация бора по данным химического анализа, мкмоль/г	Величины необратимой адсорбции для разных схем присоединения, мг/г				Преимущественная схема присоединения
		расчет из данных химического анализа			опыт	
		BCl_3	$-BCl_2$	$>BCl$		
32	56	6.6	4.6	2.6	4.9	$-BCl_2$
58	51	6.0	4.2	2.4	4.9	$-BCl_2$
100	72	8.5	5.9	3.3	4.9	$-BCl_2$, $>BCl$

Из данных химического анализа (см. таблицу) видно, что при взаимодействии трихлорида бора с алмазом реализуется в основном схема присоединения $[C]_m(OH)_{n-x}(OBCl_2)_x$.

На рентгеноэлектронных спектрах алмаза после взаимодействия с BCl_3 кроме основных пиков углерода остова [$E_{св.}$ (C1*s*) 284.9 эВ] и кислорода [$E_{св.}$ (O1*s*) 531.8 эВ] присутствуют пики бора и хлора (рис. 2). Положение пика B1*s* ($E_{св.}$ 191.6 эВ) примерно соответствует энергии связи в гидроксосоединениях бора ($E_{св.}$ 191.8 эВ) [4], что объясняется частичным гидролизом связей B—Cl водяными парами на воздухе. Пик Cl2*p* с энергией 198.8 эВ может быть отнесен к негидролизовавшимся связям B—Cl.

ЭКСПЕРИМЕНТАЛЬНАЯ ЧАСТЬ

В настоящей работе использовали препараты синтетического алмаза марки АСМ 0.3/0 и технического углерода марки ПМ-100, концентрации кислотных оксигрупп на которых составляли соответственно 2.9 и 4.0 мкмоль/м². Удельные поверхности, определенные по низкотемпературной адсорбции азота, составили 40 м²/г для алмаза и 100 м²/г для технического углерода. Взаимодействие галогенидов с препаратами углерода проводили в вакуумной гравиметрической установке [5]. Изменения веса образцов в ходе взаимодействия фиксировали с точностью ±0.1 мг при навеске исходного препарата 0.2 г. Десорбцию проводили при температуре соответствующего опыта до установления постоянного веса при остаточном давлении не более 1 мПа. Поверхностную концентрацию бора определяли колориметрически по реакции с кармином [6]; бор переводили с поверхности в раствор, смывая его с образцов раствором NaOH концентрацией 0.025 моль/л.

Рентгеноэлектронные спектры образцов после взаимодействия с галогенидами снимали на спектрометре ESCALAB-5 с использованием AlK_α-излучения. Положение пиков B1*s* и Cl2*p* определялось с точностью 0.4 эВ.

Выводы

Взаимодействие галогенидов бора с поверхностью препаратов алмаза и технического углерода при температурах 32—200 °C и давлениях 0.33—3.52 кПа происходит необратимо. Скорость адсорбции галогенидов бора описывается кинетическим уравнением для однородной поверхности. Найдены энергии активации хемосорбции и константы скорости.

ЛИТЕРАТУРА

[1] *Алесковский В. Б.* Стехиометрия и синтез твердых соединений. — Л.: Наука, 1976. 140 с.

[2] *Смирнов Е. П.* Синтез твердых соединений на основе углерда. — Направленный синтез твердых веществ: Межвуз. сб. // Под ред. Алесковского В. Б. — Л.: Изд. ЛГУ, 1983, с. 51—78.

[3] *Freeman A. G., Johnstone J. H.* — Carbon, 1971, vol. 9, N 5, p. 667—671.

[4] *Нефедов В. И.* Рентгеноэлектронная спектроскопия химических соединений. — М.: Химия, 1984. 256 с.

[5] *Краснобрыжий А. В., Смирнов Е. П., Гордеев С. К., Жуков С. Г.* — ЖПХ, 1984, т. 57, № 1, с. 76—78.

[6] *Бабко А. К., Пилипенко А. Т.* Фотометрический анализ. Методы определения неметаллов. — М.: Химия, 1974, с. 45—68.

Ленинградский технологический институт имени Ленсовета

Поступило в Редакцию 28 мая 1986 г.

Interaction of boron halides with carbon preparations

Gavrilin, A.V., Luk'yanov, I.M., Smirnov, E.P., & Aleskovskij, V.B.

Abstract. Interaction of boron trichloride and tribromide with hydroxyfunctional groups of diamond and carbon black surface with halide vapours in the pressure range of 0.33-3.52 kPa and in the temperature range of 32-200 °C has been investigated. It is shown that interaction under these conditions takes place irreversibly. The rate of boron halide adsorption is described by the kinetic equation of adsorption on a homogeneous surface. Activation energies of chemisorption and rate constants are determined.

Consideration of solid carbon substances such as diamond and graphite within the framework of "core hypothesis" **[1]** has facilitated the elucidation of the chemical structure of the macroradicals and the functional groups, leading to the resolution of several challenges pertaining to the controlled synthesis of chemically monolayer halide and oxide structures **[2]**. The literature describing the interaction of boron halides with carbon materials is scarce, with predominant focus on intercalated graphite compounds **[3]**. However, the synthesis and utilization of boron-carbon compounds using non-graphitic carbon materials presents considerable scientific and practical significance, particularly in relation to the challenges associated with procuring semiconductor diamonds and catalytic graphitization of various forms of carbon. This study investigates the interaction between boron trichloride and tribromide vapours and the oxyfunctional surface groups of diamond and carbon black at vapour pressures ranging from 0.33 to 3.52 kPa and temperatures between 32 and 200 °C.

Figure 1 presents typical non-equilibrium adsorption isotherms of different carbon materials when exposed to halide vapors. Upon conducting experiments with BCl_3, it was observed that after removing the adsorbate, there was no desorption detected. Conversely, experiments involving BBr_3 exhibited a substantial desorption rate. As the temperature of the experiment increased (as depicted in Fig. 1a), the quantity of product capable of desorption progressively decreased, a characteristic feature of physical interaction. Notably, at an experimental temperature of 200 °C, desorption was virtually nonexistent. The quantity of irreversibly adsorbed BBr_3 remained nearly constant across the range of temperatures studied (see Fig. 1a). These kinetic profiles were effectively modelled using the following equation:

$$(1-\theta) = e^{-kt}$$

Where, the parameter θ represents the extent of surface coverage at a specific time t. The rate constant k is defined as $k=k_0 \exp(-E_a/RT)$, wherein E_a stands for the activation energy, R denotes the universal gas constant, and T stands for the absolute temperature. During the analytical procedures, it was postulated that $\theta = 1$ holds true across all adsorption capacities of 4.9, 15, and 42 mg/g concerning the BCl_3-diamond, BBr_3-diamond, and BBr_3-carbon black systems, respectively. In the kinetic assessments pertaining to the BBr_3-diamond and BBr_3-carbon black specimens, sole consideration was given to

irreversible adsorption, as depicted by the conduction of intermediate desorption (Fig. 1a), facilitating the determination of the irreversible adsorption level at any given moment. Across all examined systems, the kinetics of chemisorption conform to the formula describing a homogenous surface of the carbon material.

$$W = \left(\frac{\partial(1-t)}{\partial t}\right)_{P=const} = -ke^{-kt}$$

The activation energies and the pre-exponential factors in the rate constant equation, as determined from the *lnk=f(1/T)* relationships, were found to be 18±4 kJ/mol and 21±5 s^{-1} for the BCl_3-diamond system, and 11±4 kJ/mol and 1.1±0.5 s^{-1} for the BBr_3-diamond system.

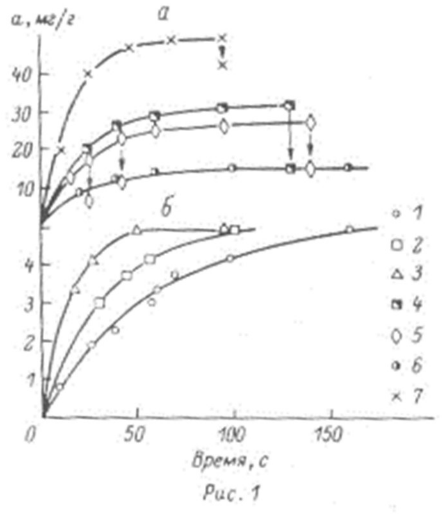

Figure 1. Non-equilibrium adsorption isotherms (adsorption capacity in mg of boron halide per g of carbon material versus time in s) of **(a)** boron bromide and **(b)** boron chloride on diamond (*1-6*) and on carbon black (*7*). Temperature (°C): *1* – 32; *2* – 58; *3,4* – 100; *5* – 150; *6,7* – 200. Pressure (kPa): *1–3* – 0.53 kPa; *4–6* – 3 kPa; *7* – 1.5 kPa. The arrows indicate desorption.

The interaction between boron halides and hydroxy groups of carbon-based compounds can be described by applying the following model:

$[C]_m (OH)_n + xBX_3 \rightarrow [C]_m(OH)_{n-x-r} (OBX_2)_{x-r}(O_2BX)_r + (x+r) HX$

where $[C]_m$ stands for the carbon macroradical and X stands for halogen (Cl or Br). Translator's note: Hl, which stood for halogen in the original version has been substituted by X, which is more common nowadays.

Upon inspection of the chemical analysis data presented in the accompanying table, it can be observed that the addition scheme involving boron trichloride and diamond predominantly manifests as $[C]_m(OH)_{n-x}(OBCl_2)_x$.

Upon subjecting diamond to interactions with BCl_3, analysis of the X-ray photoelectron (XPS) spectra revealed the presence of XPS C 1s (Binding energy (BE) of 284.9 eV) and O 1s (BE of 531.8 eV) core level bands, alongside identifiable peaks of boron and chlorine (Fig. 2).

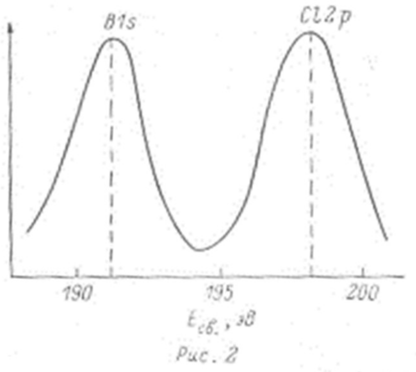

Figure 2. B 1s and Cl 2p core level XPS spectra of diamond after interaction with boron chloride.

The peak position of B 1s (BE of 191.6 eV) approximately aligns with the BE observed in boron hydroxo compounds (BE of 191.8 eV), indicating partial hydrolysis of B–Cl bonds by water vapor in the ambient air. Furthermore, the Cl 2p peak registered at a BE of 198.8 eV is attributed to non-hydrolyzed B–Cl bonds.

Temperature, °C	Surface boron concentration (based on chemical analysis), $\mu mol/g$	Irreversible adsorption values, mg/g				Predominant addition mechanism
		Based on chemical analysis data			Experimental	
		BCl_3	$-BCl_2$	$>BCl$		
32	56	6.6	4.6	2.6	4.9	$-BCl_2$
58	51	6.0	4.2	2.4	4.9	$-BCl_2$
100	72	8.5	5.9	3.3	4.9	$-BCl_2$, $>BCl$

Conclusions

In conclusion, the irreversible interaction of boron halides with both diamond and carbon black preparations was observed to take place within the temperature range of 32–200 °C and under pressures ranging from 0.33 to 3.52 kPa. The adsorption rate of boron halides was accurately described by a kinetic equation for a homogeneous surface. Additionally, the activation energies of chemisorption and the rate constants were successfully determined.
